# Rational Design of a DNA‐Scaffolded High‐Affinity Binder for Langerin

**DOI:** 10.1002/anie.202006880

**Published:** 2020-09-15

**Authors:** Gunnar Bachem, Eike‐Christian Wamhoff, Kim Silberreis, Dongyoon Kim, Hannes Baukmann, Felix Fuchsberger, Jens Dernedde, Christoph Rademacher, Oliver Seitz

**Affiliations:** ^1^ Department of Chemistry Humboldt-Universität zu Berlin 12489 Berlin Germany; ^2^ Department of Biomolecular Systems Max Planck Institute of Colloids and Interfaces 14424 Potsdam Germany; ^3^ Institute of Laboratory Medicine, Clinical Chemistry and Pathobiochemistry Charité-Universitätsmedizin Berlin corporate member of Freie Universität Berlin, Humboldt-Universität zu Berlin and Berlin Institute of Health 13353 Berlin Germany

**Keywords:** carbohydrate recognition, DNA nanotechnology, lectins, multivalent interactions, peptide nucleic acids

## Abstract

Binders of langerin could target vaccines to Langerhans cells for improved therapeutic effect. Since langerin has low affinity for monovalent glycan ligands, highly multivalent presentation has previously been key for targeting. Aiming to reduce the amount of ligand required, we rationally designed molecularly defined high‐affinity binders based on the precise display of glycomimetic ligands (Glc2NTs) on DNA‐PNA scaffolds. Rather than mimicking langerin's homotrimeric structure with a C3‐symmetric scaffold, we developed readily accessible, easy‐to‐design bivalent binders. The method considers the requirements for bridging sugar binding sites and statistical rebinding as a means to both strengthen the interactions at single binding sites and amplify the avidity enhancement provided by chelation. This gave a 1150‐fold net improvement over the affinity of the free ligand and provided a nanomolar binder (IC_50_=300 nM) for specific internalization by langerin‐expressing cells.

## Graphical abstract

Statistical rebinding and chelate binding reinforce each other and cooperatively increase the affinity for a multivalent target. The distance‐defined display of two small clusters of a glycomimetic compound on a rigid PNA‐DNA scaffold provided the first molecularly defined, nanomolar binder of the C‐type lectin receptor langerin. The high‐affinity binders are selectively internalized by langerin‐expressing cells.

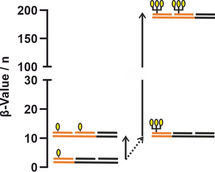

## Introduction

Carbohydrate‐protein interactions drive important biological recognition processes on cell membranes. Typically, sugar‐binding receptors such as the mammalian lectins, which govern binding of bacteria and viruses to cell membranes, have only millimolar affinity and display low specificity for monovalent carbohydrates. To increase affinity and allow specific interactions to occur at low concentration, nature takes advantage of concerted carbohydrate‐protein interactions among multivalent binding partners.^[1]^ Similarly, multivalency is the key tool for the design of compounds that target multimeric carbohydrate‐binding proteins. Impressive results have been obtained by brute force presentation of hundreds of glycan ligands on display materials such as polymers,^[2]^ liposomes,^[3]^ nanoparticles^[4]^ and carbon nanotubes.^[5]^ However, while this shotgun‐type tactic can lead to extremely high potencies the approach will reveal little information about the spatial arrangement of binding sites. An alternative approach relies on multivalent presentation on molecularly and stoichiometrically defined scaffolds such as dendrimers,^[6]^ calixarenes,^[7]^ carbohydrates,^[8]^ cyclodextrines,^[9]^ peptides^[10]^ or DNA.^[11]^

Two different interaction mechanisms guide the design of multivalent binding agents, namely the chelate effect and the statistical rebinding effect. The chelate effect is active when two or more ligands are able to bridge the carbohydrate binding sites of a multivalent receptor system. The statistical rebinding effect takes place when two or more ligands bind to only one binding site, quickly replacing each other and leading to reduced apparent off rates (Figure S1).^[12]^ Both effects can work in concert if distances between binding sites are small and glycan ligands are densely clustered or presented on short to medium length flexible scaffolds. The design criteria are more demanding for low affinity binding sites that are separated by larger distances (≥40 Å). Flexible linkers sample a large conformational space and may therefore allow both bridging of binding pockets and rebinding. However, the increase of effective molarity provided by flexible linkers may not be sufficient to reach the concentration threshold required for bridging two low affinity binding sites.^[13]^ Suitably designed rigid spacers based on capsid proteins^[14]^ or DNA^[13b]^ offer a solution to this problem. DNA‐based scaffolds are particularly attractive due to the ability to combine a small number of component strands into a wide variety of structures through sequence‐programmed hybridization. As a result, a large number of arrangements can be screened with relatively little synthetic effort.^[15]^

DNA‐type scaffolds facilitate multivalency‐enhanced binding by providing high effective molarities provided that the ligands are attached in a distance matching the distance of binding sites.^[13]^ Molecular dynamics simulations showed that nicked PNA‐DNA complexes provide rotational degrees of freedom. Bending can occur, albeit at the cost of strain that can be reduced by incorporating single stranded segments.^[15d]^ Furthermore, a substantial body of work suggests that the molecular ruler properties of DNA‐type scaffolds are not affected by the linkers used by us (meaning that highest affinities were obtained for complexes presenting the ligands in a distance that matched the distance between binding sites on structurally characterized targets).[^13b^, ^15d^, ^15e^, ^15f^, ^15g^, ^15h^, ^15i^, ^16^] However, due to the rigidity of DNA scaffolds statistical rebinding cannot contribute to the multivalent interactions if ligands are separated by large distances. Furthermore, chelation‐induced binding enhancements remain low when the strength of the monovalent interaction is weak.[^13^, ^17^]

A viable method to foster interactions with a multivalent carbohydrate‐binding protein is to increase the number of glycan ligands that simultaneously interact with the multimeric receptor system. Three‐arm DNA scaffolds have been optimized for interactions with receptors offering three binding sites.^[18]^ Such distance‐matched multivalent scaffolds are difficult to design and often the affinity enhancements remain in the order expected for bivalent interactions. Herein we propose an alternative approach to rationally improve the avidity enhancement provided by a nucleic acid scaffold.

In an exemplary study, we sought high affinity binders for the C‐type lectin langerin expressed on Langerhans cells. Langerin plays an important role in recognizing and internalizing pathogens into Langerhans cells which are able to stimulate T‐cell responses through antigen presentation.^[19]^ Therefore, targeting of langerin has the potential to allow specific delivery of vaccination agents to Langerhans cells. The langerin extracellular domain (ECD) forms a trimeric structure comprised of three carbohydrate recognitions domains (CRD).^[20]^ Rather than attempting the design of a C3‐symmetrical scaffold that would match the arrangement of langerin's sugar binding sites, we present a straightforward method to enhance the avidity of readily accessible, easy to design bivalent binders. The method hinges on the positive correlation between the strength of the monovalent ligand‐receptor interaction and the magnitude of the avidity enhancement provided by chelation.^[17]^ In this first systematic study, we use statistical rebinding to improve interactions at individual sugar binding sites and amplify the *x*‐fold chelate bivalency enhancement.

## Results and Disucssion

**Figure 1:**
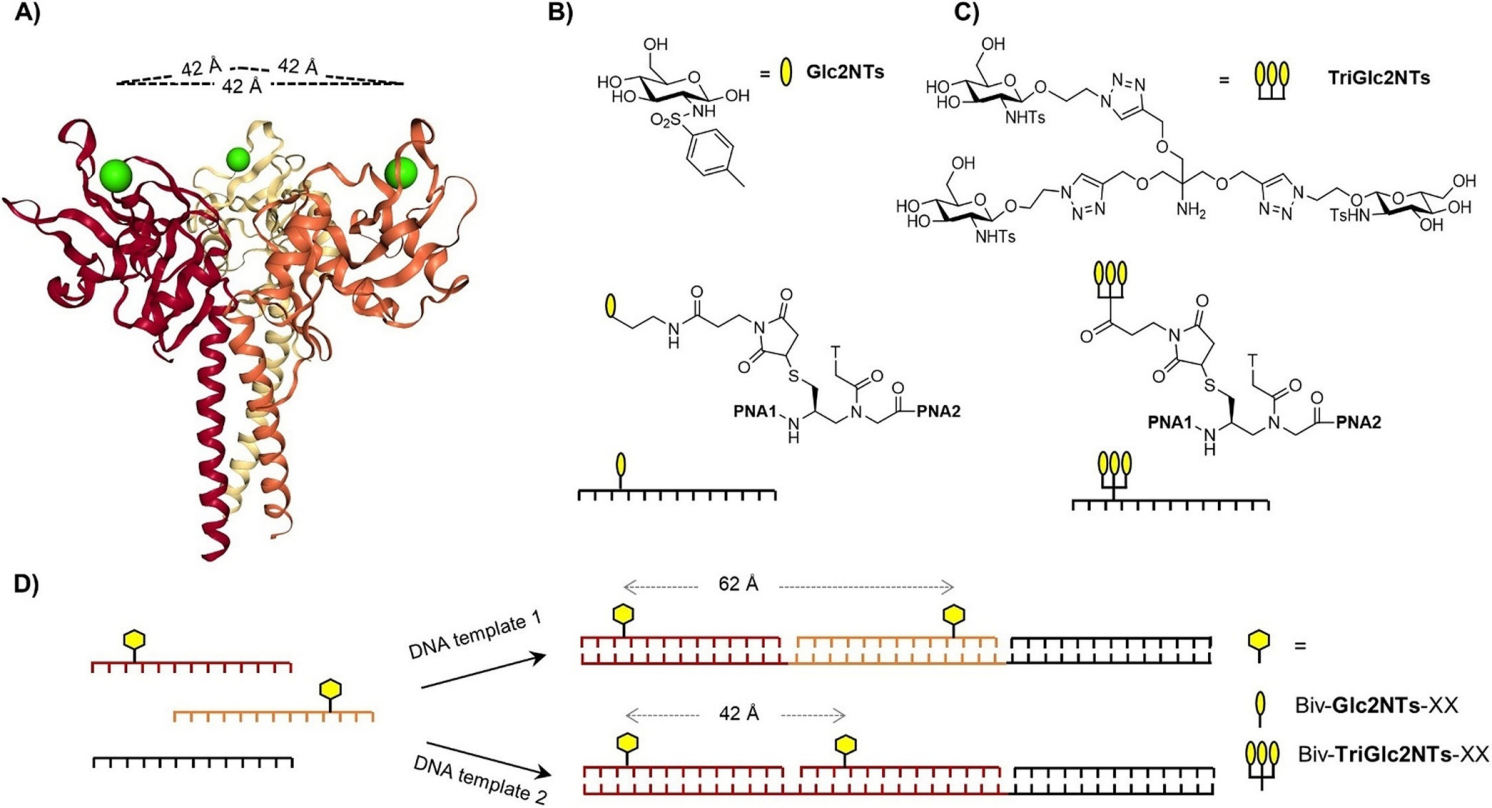
A) Crystal structure of the trimeric langerin extracellular domain (PDB ID: 3kqg) with Ca^2+^ ions (green) embedded in the binding sites, which are 42 Å apart. B) Structures of Glc2NTs ligand and Glc2NTs‐PNA conjugates. C) Structures of TriGlc2NTs ligand and TriGlc2NTs‐PNA conjugates. D) Hybridization of modified (orange, red) and unmodified (black) peptide nucleic acid (PNA) oligomers with DNA templates affords bivalent PNA‐DNA complexes displaying ligands (yellow) in different distances. PNA 1: tcatcgccttcta, PNA 2: acct*atggacttt, PNA 3: actt*acttcacgc, PNA 4: atgctacgtt*gac, PNA 5: aactcctact*cct, PNA 6: atacat*ccaacac, PNA 7: tcat*tcact*cggc, PNA 8: acct^#^atggacttt, PNA 9: atgctacgtt^#^gac (*=Glc2NTs modification; ^#^=TriGlc2NTs modification).

Crystal structure analysis showed that the carbohydrate binding sites of langerin are positioned 42 Å apart on the exposed surface of the trimer (Figure 1 A).^[20]^ In solution, the binding sites will sample the conformational space, however, at current there are no reports about the minimal and maximal possible distances accessible by the protein. Langerin recognizes a subset of high mannose oligosaccharides, blood group B trisaccharides and β‐glucans containing glucose, fucose and mannose, respectively.^[21]^ High binding affinities have been reported for sulphated saccharides.^[22]^ Potent natural monovalent ligands of langerin are N‐acetylglucoseamine (*K*_D_≈4 mM) and mannose (*K*_D_≈5 mM),[^22c^, ^23^] which are not only able to bind to langerin but also to other C‐ type lectins such as Dendritic Cell‐Specific Intercellular adhesion molecule‐3‐Grabbing Non‐integrin (DC‐SIGN) and Mannose Binding Protein.[^22a^, ^24^] Recently, we reported a specific, glycomimetic langerin ligand comprised of tosylated glucosamine (Glc2NTs, Figure 1 B), which showed submillimolar affinity (*K*_D_=0.46 mM).^[23]^ For the rational design of defined molecular systems that present Glc2NTs ligands in an arrangement allowing chelate‐enhanced binding we constructed scaffolds composed of complexes formed by Watson–Crick hybridization of a 39 nucleotide (nt) long DNA template strand with three 13 nt peptide nucleic acid (PNA) strands (Figure 1 D). In spite of the rather small length, PNA provides sufficient affinity to maintain integrity at nanomolar concentrations (Tables S1, S2).^[25]^ Two of the PNA strands carry the ligand, which was installed via 1,4 addition of thiolated PNA to maleimide‐functionalized Glc2NTs or TriGlc2NTs (Figure 1 B, C). After hybridization with the DNA template the sugar residues were spaced 5–32 nt distances apart, corresponding to 16–104 Å, based on the structure of the DNA‐PNA helical structure (Table S1, S2).^[26]^

**Figure 2:**
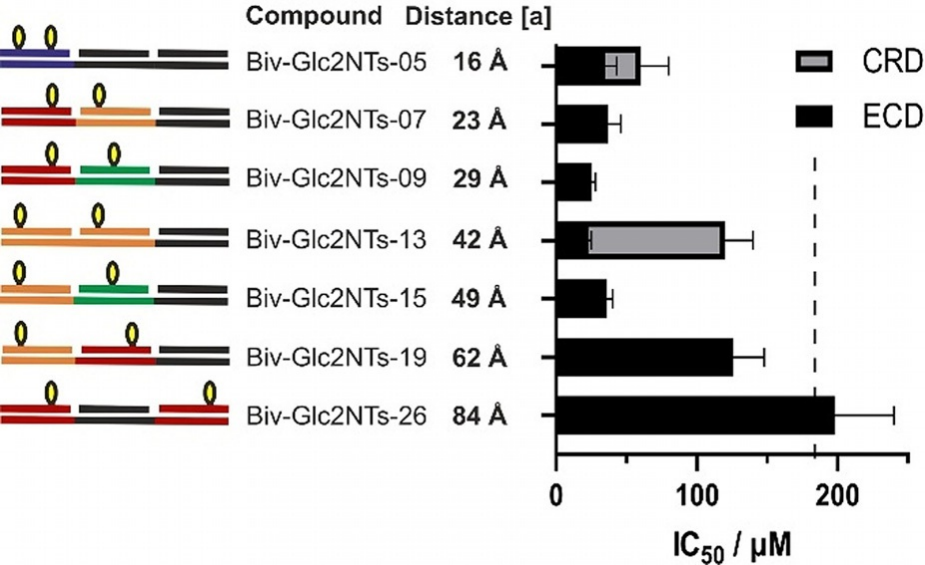
Distance‐dependent binding of bivalent Glc2NTs‐PNA‐DNA complexes to the langerin ECD assessed by a ^19^F NMR assay. IC_50_ values below the vertical dashed line (=IC_50_ (Glc2NTs)/2) can be due to bivalency‐enhanced interactions. Grey squares mark values for binding of the langerin CRD. Conditions: complexes incubated at varied concentration with langerin (50 μM or 25 μM) and 0.1 mM ^19^F‐marked reporter ligand in 25 mM Tris/HEPES with 10 % DMSO, 10 % D_2_O, 150 mM NaCl, 0.05 mM TFA and 5 mM CaCl_2_ at pH 7.8 and 25 °C. [a] Based on 3.25 Å average rise per base pair in a DNA⋅PNA duplex.

Binding to trimeric langerin ECD (hereafter named only ECD) was determined by a previously published ^19^F‐NMR reporter displacement assay.^[27]^ As expected, a high affinity was obtained for complex **Biv‐Glc2NTs‐13**, which arranges the ligands in the 42 Å distance between the CRDs of the langerin ECD (Figure 2). The IC_50_ of 23±2 μM revealed a substantial, 16‐fold bivalency enhancement over Glc2NTs (IC_50_=368 μM). Given that a 25 μM concentration of binding sites was required in the NMR experiments, this IC_50_ approaches the assay limit and the “true” IC_50_ may therefore be lower (vide infra). However, within the error of measurements the distance‐affinity profile (Figure 2) appeared rather shallow with similar IC_50_ values for complexes presenting the Glc2NTs ligands in a 16–49 Å range. A noteworthy observation is the high langerin affinity of complexes such as **Biv‐Glc2NTs‐05** that present the ligands in a distance too small (≤23 Å) to allow bridging of two langerin CRDs. Control measurements showed that the affinity loss observed for interactions with monomeric langerin CRD was substantially higher for **Biv‐Glc2NTs‐13** than for **Biv‐Glc2NTs‐05** (IC_50_ (ECD)/ IC_50_ (CRD)=5.2±0.1 for Biv‐Glc2NTs‐13 vs. 1.7±1.0 for Biv‐Glc2NTs‐05). We inferred: binding interactions provided by the 5 nucleotide long spacer in **Biv‐Glc2NTs‐05** may be enhanced by statistical rebinding, which seems facile for langerin's solvent exposed carbohydrate binding sites^[27]^ on the flat protein surface.

The system's amenability to statistical rebinding guided us to a rational design of high affinity langerin binders. Recent investigations showed that the affinity increase that can be obtained by chelate‐type multivalent interactions critically depends on the strength of the monovalent interaction.^[17]^ The stronger the interactions between individual receptor‐ligand pairs, the higher the affinity gain provided by the chelate effect. Assuming that statistical rebinding will provide an efficient means to foster the interactions with a langerin CRD and amplify the chelate‐effect we attached the trivalent glycocluster TriGlc2NTs to PNA (Figure 1 C). Though at the concentration limit, the ^19^F‐NMR reporter assay showed that trivalent TriGlc2NTs had a 30±7‐fold lower IC_50_ than the monovalent Glc2NTs, confirming that statistical rebinding can confer enhancement of langerin affinity (Table S3).

The distance‐affinity landscape suggests **Biv‐Glc2NTs‐13** as the most efficient binder of the Biv‐Glc2NTs series (Figure 2). However, given the error of the measurement such a claim is not justified. For validation, we took recourse to a surface plasmon resonance (SPR) assay (vide infra) that provided an improved IC_50_ limit of only 250 nM (compared to 12.5 μM for ^19^F‐NMR assay). The SPR assay uses a multivalent mannose‐functionalized polymer as a competitive langerin ligand. Of note, the avidity of this ligand is much higher (SPR: *K*_D_=1.5 μM) than the affinity of the competing monovalent ligand used in the ^19^F‐NMR assay (^19^F‐NMR: *K*_D_=8 mM). To obtain a conservative estimate of the SEM, the SPR signal was measured after incubating langerin (500 nM) with the ligands at a single concentration (10 μM) in three entirely independent replicates (Table S4, Figure S6). An Anova test (Suppl. Inf. page S22) confirmed that the determined differences in inhibition of the langerin—mannose‐PAA interaction are statistically significant and revealed the superior inhibitory potency of **Biv‐Glc2NTs‐13**.

To evaluate binding of avidity‐improved constructs, we used the SPR assay. The bivalent presentation of the TriGlc2NTs ligand afforded a remarkable affinity enhancement (Table 1). The net gain of affinity improved from IC_50_=347±11 μM for the monovalent Glc2NTs to IC_50_=0.3±0.02 μM for the bivalent presentation of TriGlc2NTs. Complex **Biv‐TriGlc2NTs‐13** provided a 10^2^‐fold (168±13) higher affinity than the TriGlc2NTs sugar and a 10^3^ (1156±114) times higher affinity than the free Glc2NTs ligand. The distance‐affinity profile revealed a remarkably high affinity of complex **Biv‐TriGlc2NTs‐07**, which arranges the ligands in a distance (23 Å) that appears to penalize bridging of two binding sites. However, given the slightly extended linker length of TriGlc2NTs, we believe that statistical rebinding (of a hexavalent ligand) can contribute. According to an alternative explanation, small linkers could induce langerin dimerization. However, this seems unlikely given the low protein concentration (500 nM) used. Cross‐linking should become more likely with low affinity ligands such as Glc2NTs (rather than TriGlc2NTs). However, ^19^F‐NMR experiments with **Biv‐Glc2NTs‐07** did not reveal the existence of species with rotational diffusion indicative of langerin dimers.

**Table 1:** Binding affinities of Glc2NTs, TriGlc2NTs, and the corresponding bivalent ligand‐PNA‐DNA complexes determined by SPR.

Structure	Compound	Estimated distance^[a]^	IC_50_ [μM]^[b]^
	Glc2NTs	–	347±11
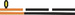	Mono‐Glc2NTs	–	105±44
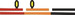	Biv‐Glc2NTs‐07	23 Å	52±2
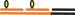	Biv‐Glc2NTs‐13	42 Å	16±1
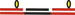	Biv‐Glc2NTs‐26	84 Å	21±2
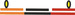	Biv‐Glc2NTs‐32	104 Å	40±14
			
	TriGlc2NTs	–	50.5±0.5
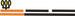	Mono‐TriGlc2NTs	–	10.5±2.2
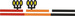	Biv‐TriGlc2NTs‐07	23 Å	0.8±0.1
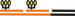	Biv‐TriGlc2NTs‐13	42 Å	0.3±0.02
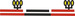	Biv‐TriGlc2NTs‐26	84 Å	1.0±0.1
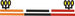	Biv‐TriGlc2NTs‐32	104 Å	1.8±0.7

[a] based on 3.25 Å average rise per base pair in a DNA⋅PNA duplex. [b] Conditions: ′5 min incubation of complexes with 500 nM langerin in 25 mM HEPES, 150 mM NaCl, 5 mM CaCl_2_, pH 7.8, 25 °C followed by determining binding of residual langerin to an α‐D‐mannose‐functionalized SPR chip.

**Figure 3:**
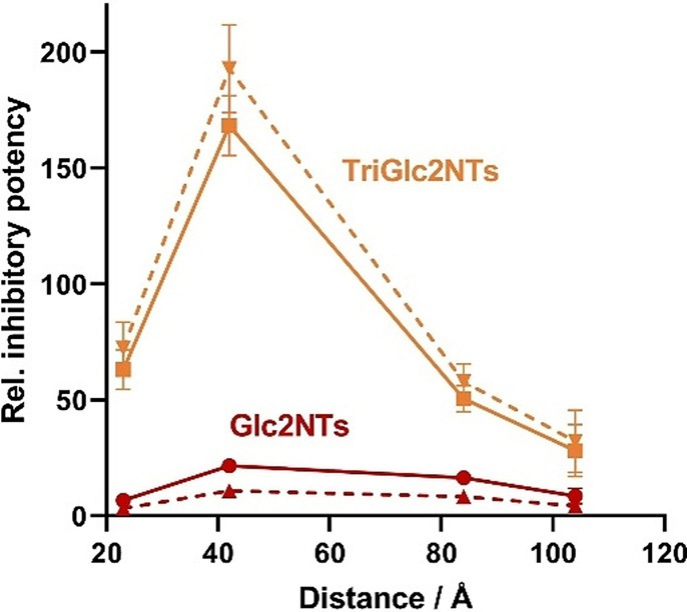
Distance dependence of relative inhibitory potency (β‐value) of bivalent complexes presenting Glc2NTs (red) or TriGlc2NTs (yellow) as multiples of the potency of unconjugated Glc2NTs and TriGlc2NTs ligands, respectively. For example, the maximum value is IC_50_(TriGlc2NTs)/IC_50_(Biv‐TriGlc2NTs‐13=168. The dashed lines represent the valency‐corrected β‐values based on the number of Glc2NTs ligands (2 ligands in Biv‐Glc2NTs, 6 ligands in Biv‐TriGlc2NTs systems). For example, the maximum valency‐corrected β‐value is IC_50_(Glc2NTs)/(6×IC_50_(Biv‐TriGlc2NTs‐13))=193. Error bars consider the propagation of errors given in Table 1.

It is instructive to compare the magnitude of the chelate‐induced binding enhancements. A graphical presentation of the distance dependence of the relative inhibitory potency illustrates that the TriGlc2NTs ligand amplifies the distance‐affinity response of the bivalent display (Figure 3). Bivalent versus unconjugated presentation of TriGlc2NTs afforded an up to 10^2^‐fold (168±13) avidity enhancement, whereas the weaker ligand Glc2NTs allowed only 10^1^‐fold (22±2) stronger binding. We conclude that statistical rebinding can be exploited to increase the chelate effect in the same way that a higher monovalent ligand affinity can be utilized to increase the avidity of a multivalent binding agent.

**Figure 4:**
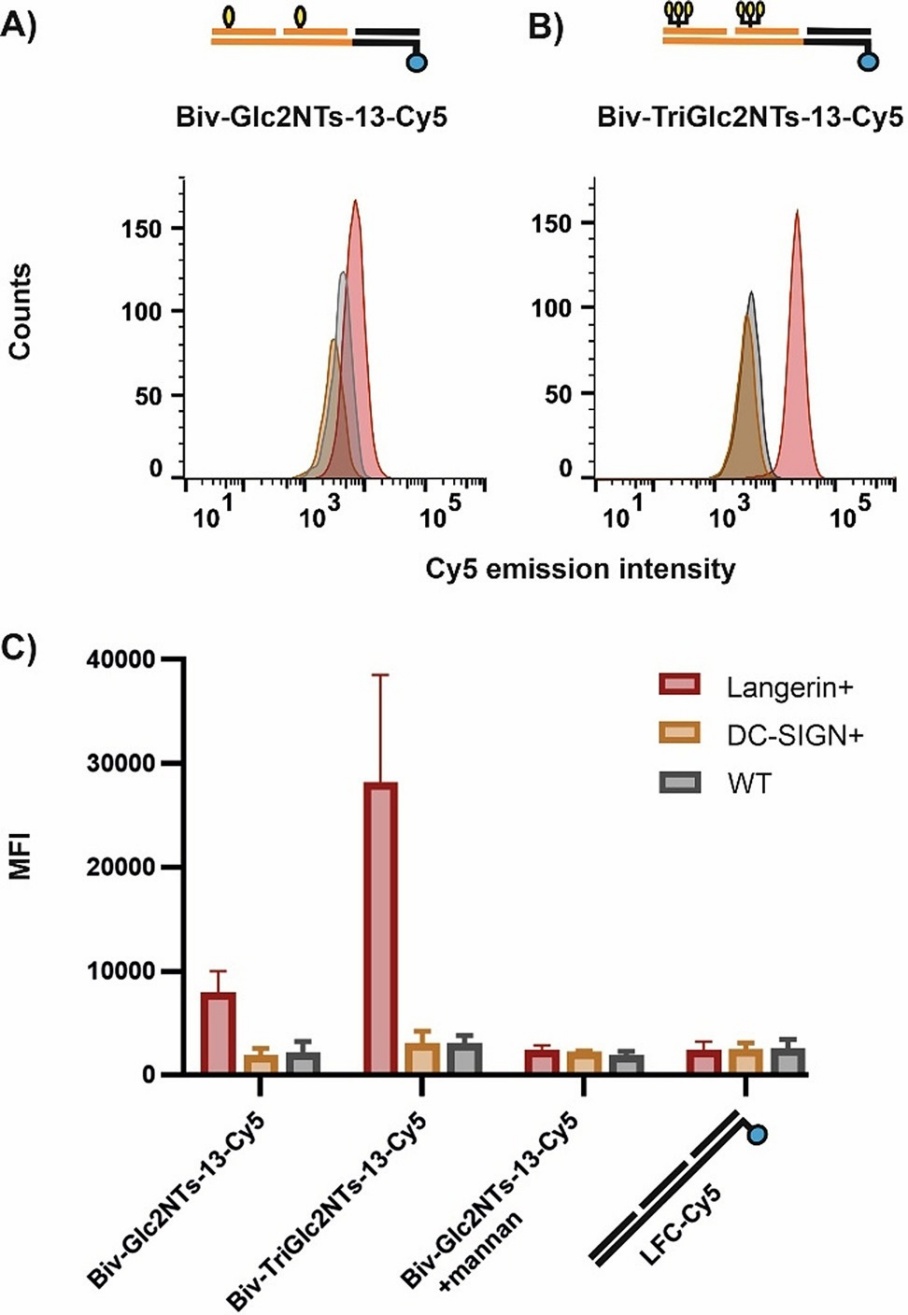
Biv‐Glc2NTs‐13 and Biv‐TriGlc2NTs‐13 target Raji cells expressing langerin but not wildtype Raji cells or Raji cells expressing DC‐SIGN. A, B) Histogram presentation of flow cytometry data from cells incubated with Cy5‐labelled Biv‐Glc2NTs‐13 (A) and Biv‐TriGlc2NTs‐13 (B). C) Mean fluorescence intensity (MFI) of cells after subtraction of autofluorescence background binding/internalization of Cy5 labelled Biv‐Glc2NTs‐13, Biv‐TriGlc2NTs‐13 and ligand‐free complex LFC‐Cy5. Conditions: Cells were incubated with complexes (660 nM) in cell media for 45 min at 4 °C and after media exchange, incubated for 60 min at 37 °C.

Next, we evaluated binding and internalization of the avidity‐enhanced assemblies by langerin‐expressing cells. Displays presenting the Glc2NTs and TriGlc2N2Ts ligands in 42 Å distance were assembled by using Cy5‐labeled DNA templates and incubated with Raji cells at 660 nM concentration. Flow cytometry measurements revealed that **Biv‐TriGlc2NTs‐13** selectively targeted langerin‐expressing Raji cells (Figure 4 B). The mean fluorescence intensity of cells expressing langerin was 9 times higher than of wild‐type Raji cells (Figure 4 C). With the lower affinity ligands **Biv‐Glc2NTs‐13** under identical conditions, staining was weaker and langerin^+^ Raji cells showed only a 4 times higher fluorescence intensity than wild‐type Raji cells. Fluorescence microscopic analysis confirmed cell uptake of the bivalent probe (Figure S9). Targeting was specific for langerin^+^ cells. Cells expressing DC‐SIGN (a C‐type lectin also binding mannose and fucose type carbohydrates) were not stained. Furthermore, addition of the strong canonical langerin inhibitor mannan prevented staining. As a further control, cells were incubated with a ligand‐free DNA‐PNA complex. We concluded that the display systems bind to the cells canonically via the langerin receptor and the PNA‐DNA scaffold does not bind unspecifically.

High affinity probes can reduce unspecific binding as they allow for the use of decreased probe concentrations, which eliminates the need for washing steps. Staining of the Raji cells was repeated with 66 nM **Biv‐Glc2NTs‐13** and **Biv‐TriGlc2NTs‐13** without washing after incubation with the compounds. Indeed, with TriGlc2NTs display **Biv‐TriGlc2NTs‐13** staining of langerin^+^ Raji cells succeeded whereas staining was inefficient with the lower affinity bivalent probe **Biv‐Glc2NTs‐13** (Figure S8).

## Conclusion

Langerin binds a variety of glycans containing mannose, fucose and sulfated sugars.[^21^, ^22c^] Given this rather broad substrate range and considering the multitude of other lectins such as DC‐SIGN, dectin‐2 and Mannose Binding Protein binding similar structures, it has been difficult to selectively target langerin‐expressing cells. Recently, we introduced the glycomimetic Glc2NTs moiety as a selective ligand and we demonstrated that attachment of Glc2NTs to liposomes provided vehicles for targeting of langerin.^[23]^ However, display systems based on polymers,[^28^, ^29^] or liposomes carry tens to thousands of glycan ligands and only a few will be involved in the recognition process. It was the aim of the work described herein to provide non‐polymeric, molecularly well‐defined high affinity langerin binders that require only a few glycomimetic ligands. Similar attempts have recently been reported by the Hartmann and Taniguchi groups. Taniguchi and colleagues arranged 6‐sulfo‐Galβ4(6‐sulfo‐GlcNAc) disaccharides in a C_3_‐symmetric fashion and reported an IC_50_=2.7 μM (compared to IC_50_=3.5 mM for the disaccharide) by applying an ELISA‐type assay with immobilized langerin.^[29]^ An oligomerized form presenting 33 copies on average provided for 10^6^‐fold improvement of ligand potency. The influence of surface loading on affinity was not addressed. Hartmann et al. used artificial 3‐arm oligoamides for presentation of three mannose units. The applied solution‐phase ^19^F‐NMR assay (vide supra) showed an IC_50_=44±6 μM for the best binding system providing a 10^2^‐fold (102±25) affinity increase compared to the free ligand.^[30]^ For comparison, the presentation of the Glc2NTs ligand on the self‐assembled DNA‐PNA scaffolds allowed for 10^3^‐fold (1156±114) enhancement of the ligand affinity and afforded binders with nanomolar affinity (IC_50_=0.3 μM).

The high affinity provided by constructs such as **Biv‐TriGlc2NTs‐13** rely on i) the use of the recently discovered Glc2NTs ligand that has one order of magnitude higher affinity for langerin than the canonical mannose ligand, and ii) a careful design allowing the combination of statistical rebinding to strengthen the interaction with a single langerin CRD and chelate‐enhanced binding. The alliance of chelation and statistical rebinding bears resemblance to an approach reported by Bernardi at al.^[31]^ They targeted DC‐SIGN rather than langerin and arranged small trivalent clusters of modified mannose bivalently on rigid rod‐like scaffolds comprised of phenylene‐ethylene units. It is instructive to compare the results since both C‐type lectin receptors have rather flat binding surfaces and the monovalent ligands bind their respective targets with similar affinity. Bernardi reported an IC_50_=5 μM for optimal scaffolds, which based on the affinity of the free ligand (IC_50_=270 μM) for DC‐SIGN corresponds to a relative potency per ligand=9. The spatial screening of langerin with DNA‐PNA scaffolds exposed that bivalent presentation of trivalent clusters can provide for an order of magnitude enhanced relative potency per ligand=193, which demonstrates the power of high precision display from DNA‐type scaffolds.

A more detailed analysis points to the factors contributing to the 1156±114‐times net improvement of affinity (193±19‐fold improvement of relative affinity per ligand). Statistical rebinding introduced by the trivalent cluster accounts for a 2.3±0.1‐fold enhancement of relative potency per ligand (IC_50_ (TriGlc2NTs)=50.5±0.5 μM vs. IC_50_ (Glc2NTs)=347±11 μM). Attachment of the ligand system to PNA provides a further 4.8±1‐fold enhancement of relative potency (IC_50_ (Mono‐TriGlc2NTs)=10.5±2.2 μM vs. IC_50_ (TriGlc2NTs)=50.5±0.5 μM). The improved affinity upon attachment to DNA scaffolds has also been seen in investigations of other carbohydrate binding proteins.[^13b^, ^15g^, ^32^] As shown in control experiments, unspecific binding of the DNA‐PNA scaffold seems unlikely and we attribute this affinity increase to steric shielding effects.^[33]^ A comparison of the monovalent and bivalent display systems exposes the contribution of chelate‐enhanced binding. This effect affords a 17.5±4.8‐fold improvement of relative affinity per ligand ((IC_50_ (Biv‐TriGlc2NTs‐13)=0.3±0.02 μM vs. IC_50_ (Mono‐TriGlc2NTs)=10.5±2.2 μM) and moves the net affinity to the nanomolar range. This detailed analysis also points to the importance of the strength of interactions at individual binding sites. With the low affinity Glc2NTs ligand chelation allowed only for a 3.3±0.2‐fold improvement of relative affinity per ligand, which was increased to 17.5±4.8‐fold with the higher avidity ligand TriGlc2NTs. Our results support that the strength of the monovalent receptor‐ligand interaction plays a key role in the affinity gain achievable by bivalent presentation. However, identifying better monovalent ligands can be challenging. We present statistical rebinding as an easy to implement alternative approach to improve chelation‐induced affinity enhancements, with particular importance for challenging target classes such as lectins. While we focused this study on the bivalent presentation of monovalent and trivalent ligands, it seems possible that higher valencies could further improve affinity by statistical rebinding. However, a higher number of clustered ligands may lead to competitive steric shielding, thereby limiting the positive influence of ligand quantity on statistical rebinding.

In summary, the strategy of combining chelate‐binding and statistical rebinding of a glycomimetic compound on rigid PNA‐DNA scaffolds provided the most potent, molecularly defined langerin binder to date, highlighting the usefulness of scaffolds that allow a precise tuning of ligand‐ligand distances. The high affinity binders were internalized by Raji cells expressing langerin but not by cells expressing DC‐SIGN or wild‐type Raji cells. DNA hybridization provides for facile attachment of cargo. While we focused on appending a fluorescent label in this work, we envision other types of cargo such as vaccination agents or a cytotoxic agent being similarly attached. Beyond recognition of C‐type lectins, we foresee interesting applications of DNA‐type display systems for the recognition of specific cell types.

## Conflict of interest

The authors declare no conflict of interest.

## Supporting information

As a service to our authors and readers, this journal provides supporting information supplied by the authors. Such materials are peer reviewed and may be re‐organized for online delivery, but are not copy‐edited or typeset. Technical support issues arising from supporting information (other than missing files) should be addressed to the authors.

Supplementary
