# Supplementary material for: Rational Design of a DNA‐Scaffolded High‐Affinity Binder for Langerin
Source: Angew Chem Int Ed Engl. 2020 Sep 15;59(47):21016–22. doi: 10.1002/anie.202006880 (PMC7693190; doi:10.1002/anie.202006880)
Supplement: Supplementary file 1 — Supplementary [file ANIE-59-21016-s001.pdf]

Angewandte Chemie
Eine Zeitschrift der Gesellschaft Deutscher Chemiker

Supporting Information

Rational Design of a DNA-Scaffolded High-Affinity Binder for Langerin

*Gunnar Bachem, Eike-Christian Wamhoff, Kim Silberreis, Dongyoon Kim, Hannes Baukmann, Felix Fuchsberger, Jens Dernedde, Christoph Rademacher, and Oliver Seitz**

anie_202006880_sm_miscellaneous_information.pdf

Table of content

1. **Chelate Effect vs Statistical Rebinding Effect** 2
2. **General Information** 2
3. **Synthesis of Glc2NTs** 3
5. **Synthesis of TriGlc2NTs** 5
7. **Synthesis of Mercaptomethylated PNA monomer** 7
8. **Synthesis of Glc2NTs-PNA conjugates** 8
9. **Ligand-PNA-DNA complexes** 15
10. **Langerin ECD and CRD Receptor Expression and Purification** 17
11. **^{19}F-NMR Assay Inhibition Experiments** 17
12. **SPR Assay Inhibition Experiments** 20
13. **C-type Lectin+ Model Cells** 24
14. **Flow Cytometry Assay** 24
15. **Confocal Microscopy of COS-7 cells** 26

1. Chelate Effect vs Statistical Rebinding Effect

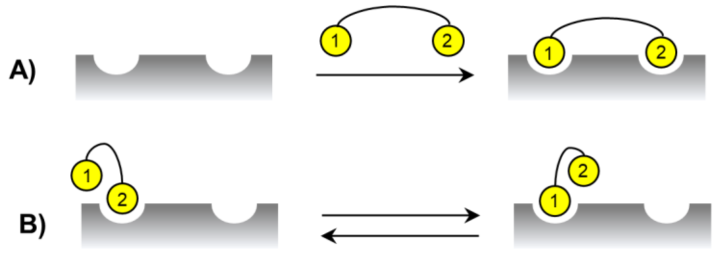

Figure S1. A) Chelate Effect: Bivalent Binder bridges two binding sites of a bivalent receptor B) Statistical Rebinding effect: Two identical ligands (yellow) of a bivalent binder interact with the same binding site of a trimeric receptor, quickly replacing each other.

2. General Information

PNA monomers were purchased from LGC LINK (Strathclyde, UK). Fmoc-protected lysine and Fmoc-protected aspartate were obtained from Novabiochem (Schwalbach, Germany). HCTU was purchased from Carl Roth (Karlsruhe, Germany). HOBt was obtained from Angene (Nanjing, China), DMF (low in water grade) was purchased from VWR (Darmstadt, Germany). DNA (HPLC-purified) was purchased from Biomers (Ulm, Germany). All other chemicals were provided from Acros Organics (Geel, Belgium), Sigma-Aldrich (Schnelldorf, Germany) and Merck (Darmstadt, Germany). Water was purified with a Milli-Q Ultra-Pure Water Purification System from Merck. (1-Thyminyl)-acetic acid [1], 2 *N*-succinimidyl-3-maleimidopropionate [2], mercaptomethylated PNA monomer [3], 2-aminoethyl 2-deoxy-2-(*p*-toluenesulfonylamido)-β-D–glucoside,[4] tris[(propargyloxy)methyl]aminomethane[5] and propargyl-2-deoxy-2-trifluoroacetamido-α-mannoside [4] (reporter ligand) were synthesized as previously described.

Column chromatography was performed with SDS 60 ACC silica gel. Silica gel 60 F254 aluminum sheets from Merck were used for thin layer chromatography.

Melting temperatures were measured on a Varian Cary Bio 100 UV-Vis spectrometer or Jasco V-750 spectrometer.

1H- and 13C-NMR spectra were measured with an AVANCE II 400, Avance II 500 MHz spectrometer (Bruker) or 600 MHz spectrometer (Agilent). The signals of the protonated solvents were used as reference signals. Chemical shifts are given in ppm (parts per million). ^{19}F R2-filtered NMR experiments were conducted on a PremiumCompact 600 MHz spectrometer (Agilent). Spectra were processed in MestReNova and data analysis was performed with Origin .[6]

Analytical HPLC was carried out on the UPLC-MS Waters ACQUITY UPLC System Qda as mass detector (column: ACQUITY UPLC BEH C18 1.7 µm) and solvents A (98.9% H2O, 1% acetonitrile, 0.1% TFA) and solvents B (98.9% acetonitrile, 1% H2O, 0.1% TFA) in a linear gradient with a flow rate of 0.5 mL/min at 50°C.

An Agilent 1100 series instrument was used to perform **semi-preparative HPLC** (column: Varian Polaris C18-A, 250 x 10.0 mm) with a flow rate of 6.0 mL/min and **preparative HPLC** (column: Macherey-Nagel VP250/21 C18 Nucleodur Gravity, 250 mm x 21 mm, 5 µm) with a flow rate of 15 mL/min, with solvents A (98.9% H_2O, 1% acetonitrile, 0.1% TFA) and solvents B (98.9% acetonitrile, 1% H_2O, 0.1% TFA) in a linear gradient. Nagel VP250/21 C18 Nucleodur Gravity, 250 mm x 21 mm, 5 µm) with a flow rate of 15 mL/min, with solvents A (98.9% H_2O, 1% acetonitrile, 0.1% TFA) and solvents B (98.9% acetonitrile, 1% H_2O, 0.1% TFA) in a linear gradient.

Concentrations of PNA and DNA oligomers were determined by measuring the optical density by using a NanoDrop ND-1000 Spectrophotometer. Molar extinction coefficients for the DNA oligomers at 260 nm were calculated with the OligoAnalyzer from Integrated DNA Technologies by the nearest neighbor method (http://eu.idtdna.com/calc/analyzer). For the PNA oligomers the molar extinction coefficients at 260 nm of the used PNA monomers were calculated via the PNA tool from PNA Bio (https://www.pnabio.com/).The absorption of the carbohydrate ligands and the amino acid residues were neglected.

3. Synthesis of Glc2NTs

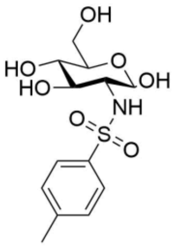

2-Deoxy-2-tosylamido-D-glucopyranose (1)
According to a literature procedure [7] *p*-toluenesulfonyl chloride (13.9 mmol, 2.65 g, 1 eq.) was dissolved in 15 mL acetone, added to glucoseamine hydrochloride (13,9 mmol, 3 g, 1 eq.) in 30 mL 1M NaOH and stirred for 4 hrs. The solvent was removed under reduced pressure and the residue purified by flash chromatography (methanol:dichloromethane 1:5) to yield the product as a white powder (4.1 g, 12.51 mmol, 90 %). The ratio of α- and β-anomer was determined to be 10:1 via ^{1}H NMR. Here, only chemical shifts corresponding to the β-anomer are documented. R_f = 0.38 with 9:1 dichlormethane:methanol.
^{1}H NMR (400 MHz, MeOD, β-anomer): δ = 7.82 – 7.77 (m, 2 H, Ph-H), 7.37 – 7.32 (m, 2 H, Ph-H), 4.77 (d, J = 3.5 Hz, 1 H , 1-CH), 3.76 – 3.69 (m, 2 H, 6-CHa, 5-CH), 3.68 – 3.55 (m, 2 H, 6-CH6, 3-CH), 3.30 – 3.24 (m, 1 H 4-CH), 3.09 (dd, J = 10.1, 3.6 Hz, 1 H, 2-CH), 2.41 (s, 3 H, OCH_3). ^{13}C NMR (100.6 MHz, MeOD, β-anomer): δ = 144.4 (1 C, C_q), 140.4 (1 C, C_q), 130.6 (2 C, Ph-H), 128.1 (2 C, Ph-H), 92.9 (1 C, 1-CH), 72.8 (1 C, 5-CH), 72.5 (1 C, 3-CH), 72.2 (1 C, 4-CH), 62.6 (1 C, 5-CH); 59.8 (1 C, 2-CH), 21.5 (1C, OCH_3).

4. Synthesis of Maleimido-Glc2NTs

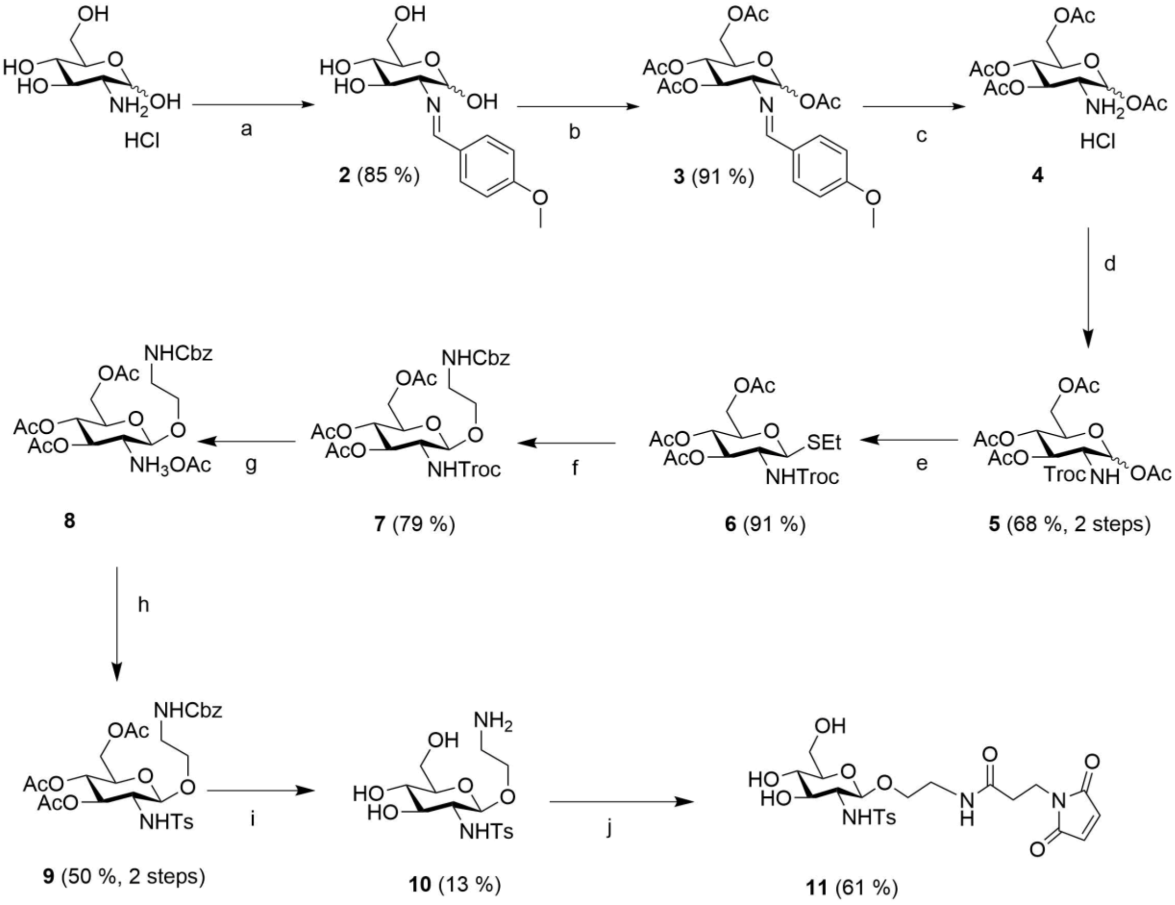

Scheme S1. Intermediate 10 was prepared as previously described.[4] Reaction conditions: a) *p*-anisaldehyde, 1 M aqueous sodium hydroxide, 0 °C; b) acetic anhydride, pyridine 0 °C to rt; c) 5 M aqueous hydrochloric acid reflux; d) trichloroethyl chloroformate, pyridine, 0°C; e) ethanethiol, $BF_3 \cdot OEt_2$, dichloromethane; f) N-Cbz-ethanol amine, DMTST, DCM, rt; g) Zn, acetic acid, rt; h*p*-toluenesulfonyl chloride, pyridine, rt; i) 1) sodium methoxide, methanol, rt; 2) H_2/Pd, ethanol, rt; j) 3-(maleimido)propionic acid NHS ester, sodium hydrogen carbonate, dioxane/water, rt

(N-(3‘-Maleimidopropanoyl)--2-aminoethyl-2-N-(*p*-toluenesulfonyl) - β-D – glucosepyranoside (11)

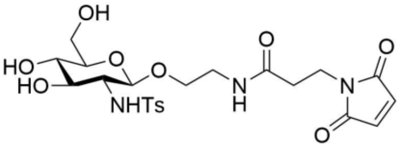

(Maleimido-Glc2NTs)

2-Aminoethyl2-deoxy-2-(*p*-toluenesulfonylamido)-β-D–glucoside **10**[4] (0.24 mmol, 90,2 mg, 1 eq., 0.1M) was dissolved in 2.4 mL deionised water and added to a solution of N-succinimidyl-3-maleimido-propionat (0.36 mmol, 95, 8 mg, 1.5 eq., 0.1 M dioxane). Sodium hydrogen carbonate solution (0.1M, 2.4 mL) was added and left to shake for 1 h. After UPLC control showed completion of the reaction 5 mL water was added, the solvent removed via lypholisation and the remaining compound purified by preparative HPLC (03 % to 50 % B in A within in 30 min). Subsequent lypholisation of the product fractions gave the title maleimide as a white powder (146 umol, 77 mg, 61 %). ^{1}H NMR (500 MHz, MeOD) δ = 7.78 (d, *J* = 8.3

Hz, 2H, Ph-*H*), 7.32 (d, *J* = 8.0 Hz, 2H, Ph-*H*), 6.80 (s, 2H, CH=CH), 4.22 (d, *J* = 8.3 Hz, 1H, 1-CH), 3.84 (dd, *J* = 11.8, 2.2 Hz, 1H, CH_{2a}-OH), 3.77 (td, *J* = 6.9, 1.3 Hz, 2H, O-CH_2-CH_2-NH), 3.66 – 3.61 (m, 2H, CH_{2b}-OH, CH_{2a}-CH_2-NH), 3.29– 3.05 (m, 7H, CH_{2b}-CH_2-NH, Cq-CH_2-CH_2-N, 2CH, 3-CH, 4-CH,5-CH), 2.47 (t, *J* = 6.9 Hz, 2H, Cq-CH_2-CH_2-N), 2.41 (s, 3H, OCH_3). ^{13}C NMR (126 MHz, MeOD) δ 173.13 (1C, $C_{q,}$, Maleimide), 172.24 (1C, $C_{q,}$ Maleimide), 144.21 (1C, $C_{q,,}$ Ph), 141.09 (1C, $C_{q,,}$ Ph), 135.54 (2C, CH=CH), 130.26 (2C, Ph-H), 128.27 (2C, Ph-H), 103.33 (1C, 1-CH), 77.80 (1C, CH), 76.43 (1C, CH), 72.13 (1C, CH), 69.45 (1C, O-CH_2), 62.67 (1C, 6-CH_2), 61.27 (1C, CH), 40.32 (1C, CH_2), 35.66 (3C, CH_2, CH_2, NH-C_q-CH_2), 21.47 (1C, OCH3).

5. Synthesis of TriGlc2NTs

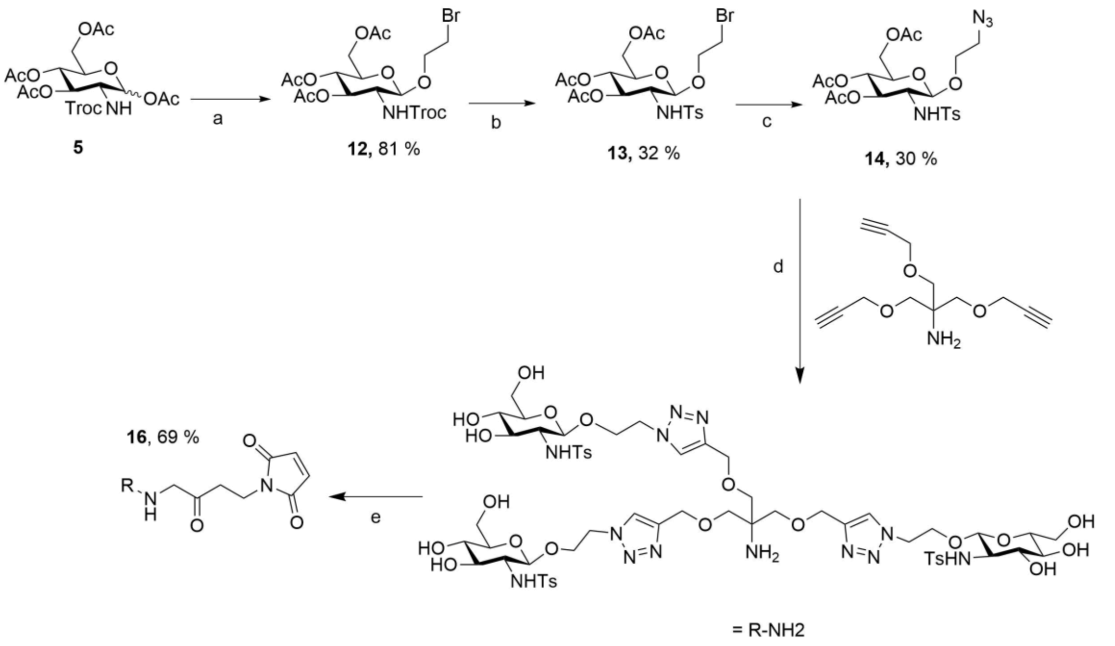

Scheme S2. Intermediate 12 was prepared as previously described.[8] Reaction conditions: a) 2-bromoethanol, $BF_3 \cdot OEt_2$, CH_2Cl_2, 0 °C to rt; b) 1) Zn, acetic acid, rt; 2) *p*-toluenesulfonyl chloride, pyridine, rt; c) sodium azide, DMF, 50 °C; d) $CuSO_4$, Tris(3-hydroxypropyltriazolylmethyl)amine, sodium ascorbate, water, 55 °C; e) 3-(maleimido)propionic acid, HATU, Et_3N, DMF, rt.

2-Bromooethyl 3,4,6-tri-O-acetyl-2-deoxy-2-(*p*-toluenesulfonylamido)-β-D-glucoside (13)

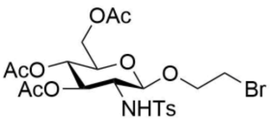

A suspension of the N-Troc protected bromoethyl glycoside **12[8]** (2 mmol, 1.1 g, 1 eq.) and freshly activated zinc (260 mmol, 20 6, 130 eq.) in 60 mL acetic acid was stirred for 4 h. The reaction mixture was filtered over celite and dried *in vacuo* to yield a white solid. Under argon the solid (1 mmol, 1.41 g, 1 eq.) was dissolved in 15 mL pyridine. 4-Toluenesulfonyl chloride (6 mmol, 1.14 g, 6 eq.) and powder molecular sieve were added. The reaction mixture was left to stir overnight, the solvent removed *in vacuo* and the residue purified by flash chromatography (1:1 cyclohexane:ethyl acetate) to yield the desired product as a white powder. (0,32 mmol, 170 mg, 32 %) ^{1}H NMR (500 MHz, $CDCl_3$) δ 7.75 (d, *J* = 8.3 Hz, 2H, Ph-H), 7.27 (d, *J* = 7.9 Hz,

2H, Ph-H), 5.40 (d, *J* = 8.5 Hz, 1H, NH), 5.07 (m, 2H, 3-CH, 4-CH), 4.42 (d, *J* = 8.2 Hz, 1H, 1-CH), 4.23 (dd, *J* = 12.3, 4.9 Hz, 1H, 6-CHa), 4.08 (dd, *J* = 12.3, 2.3 Hz, 1H, 6-CHb), 3.87 – 3.79 (m, 1H, CH2a-Br), 3.66 (m, 1H, 5-CH), 3.52 (m, 2H, CH2b-Br, 2-CH), 3.09 (ddd, *J* = 10.2, 7.4, 5.5 Hz, 1H, CH_{2a}-CH_2-Br), 3.00 (ddd, *J* = 10.2, 7.4, 6.8 Hz, 1H, CH_{2b}-CH_2-Br), 2.41 (s, 3H, OCH_3), 2.07 (s, 3H, OAc), 2.01 (s, 3H, OAc), 2.00 (s, 3H, OAc).^{13}C NMR (126 MHz, $CDCl_3$) δ 143.68 (1C, Cq), 138.73 (1C, Cq), 129.72 (2C, Ph-H), 127.76 (2C- Ph-H), 101.89 (1C, 1-CH), 73.20 (1C, 3-CH), 72.22 (1C, 5-CH), 70.03 (1C, CH_2-Br), 68.65 (1C, 4-CH), 62.30 (1C, 6-CH_2), 58.43 (1C, 2-CH), 29.50 (1C, *C*H_2-CH_2-Br), 21.87 (3C, OCH_3), 21.13 (6C, 2xOAc), 20.99 (3C, OAc).

2- Azidoethyl-N-(*p*-toluenesulfonyl)-β-D-glucoseamide (14)

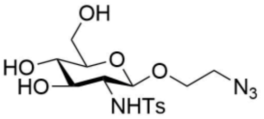

A suspension of the bromoethyl 2-deoxy-2-(p-toluenesulfonylamido)-β-D-glucoside **13** (0.32 mmol, 170 mg, 1 eq) and sodium azide (1,8 mmol, 342 mg, 6eq) in 20 mL DMF was stirred at 50°C for 22 h. Subsequently, 100 mL ethylacetate was added and the mixture washed with 0.1M HCl. After drying with magnesium sulfate, the solvents were removed in vacuo. The residue was dissolved in 10 mL methanol and sodium methanolate solution (1.6 mmol, 86.4 mg, 0.4 M, 5 eq.) was added. After 1hr UPLC control showed full conversion, the solvent was removed in vacuo and the residue purified by (HPLC 3 % to 50 % B in A within 30 min) to afford the desired product as a white powder (0.1 mmol, 40 mg, 30%). ^{1}H NMR (500 MHz, MeOD) δ 7.78 (d, *J* = 8.3 Hz, 2H, Ph-H), 7.36 (d, *J* = 8.5, 2H, Ph-H), 4.25 (d, *J* = 8.3 Hz, 1H, 1-CH), 3.87 (dd, *J* = 11.9, 2.3 Hz, 1H, 6-CH2a), 3.70 (ddd, *J* = 11.0, 6.8, 4.4 Hz, 1H, CH_{2a}-CH_2-N_3), 3.66 (dd, *J* = 12.0, 6.0 Hz, 1H, 6-CH_{2b}), 3.38 – 3.20 (m, 4H, 5-CH,4-CH.3-CH, CH_{2b}-CH_2-N3), 3.13 (dd, *J* = 9.8, 8.3 Hz, 1H, 2-CH), 3.06 (ddd, *J* = 13.0, 6.2, 4.4 Hz, 1H, CH2-CH_{2a}-N3), 2.95 (ddd, *J* = 13.0, 6.8, 4.5 Hz, 1H, CH2-CH_{2b}-N3), 2.45 (s, 3H, OCH_3). ^{13}C NMR (126 MHz, MeOD) δ 143.97 (1C, Cq), 141.46 (1C, Cq), 130.27 (2C, Ph-H), 128.21 (2C, Ph-H), 103.07 (1C, 1-CH), 77.91 (1C, 4-CH), 76.72 (1C, 3-CH), 71.99 (1C, 5-CH), 68.86 (1C, *C*H_2-CH_2-N_3), 62.75 (1C, 6CH_2-OH), 61.46 (1C, 2-CH), 51.48 (1C, CH_2-N_3), 21.45 (1C, OCH_3).

TriGlc2NTs (15)

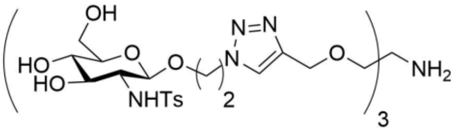

The azidoethylglycoside **14** (90 µmol, 36 mg, 4 eq) and tris[(propargyloxy)-methyl]aminomethane [5] (22.5 µmol, 7.8 mg, 1 eq) were dissolved in 500 µL water. A solution of tris(3-hydroxypropyltriazolylmethyl)amine (4.5 µmol, 2 mg, 0.2 eq), copper sulfate monohydrate (2.25 µmol, 0.5 mg, 0.1 eq) and sodium ascorbate (9 µmol, 2.8 mg, 0.4 eq) in 300 µL water was added and the reaction mixture shaken at 55°C for 24 h. After UPLC control showed full alkyne conversion, sodium hydrosulfide was added to precipitate copper. The supernatant obtained after centrifugation was purified by preparative HPLC (10 % to 40 % B in A within in 30 min) to yield the desired trivalent sugar (15.2 µmol, 22 mg, 67 %). ^{1}H NMR (500 MHz, MeOD) δ 8.08 (s, 3H, CH of triazole), 7.67 (d, *J* = 8.3 Hz, 6H, Ph-H), 7.22 (d, *J* = 8.0 Hz, 6H, Ph-H), 4.59 (d, *J* = 2.9 Hz, 6H, CH_2-Cq-N=N), 4.47 (ddd, *J* = 14.4, 7.4, 3.1 Hz, 3H, O-CH_2-CH_{2a}-N), 4.35 (ddd, *J* = 14.4, 6.1, 3.1 Hz, 3H, O-CH_2-CH_{2b}-N), 4.28 (d, *J* = 8.2 Hz, 3H 1-CH), 4.03 (ddd, *J* = 11.0, 6.1, 3.1 Hz, 3H, O-CH_{2a}-CH_2-N), 3.84 (dd, *J* = 11.9, 2.1 Hz, 3H, 6-CHa), 3.67 (s, 6H, H_2N-Cq-C*H_2*-O), 3.64 (dd, *J* = 11.9, 5.6 Hz, 3H, 6-CHb), 3.53 (ddd, *J* = 10.8, 7.4, 3.1 Hz, 3H, O-CH_{2b}-CH_2-N), 3.29 – 3.14 (m, 12H, 2-CH,3-CH, 4-CH, 5-CH),

2.36 (s, 9H, OCH_3). ^{13}C NMR (126 MHz, MeOD) δ 144.73 (3C, Cq of triazole), 144.01 (3C, C_q), 141.42 (3C, C_q), 130.25 (6C, Ph-H), 128.04 (6C, Ph-H), 126.86 (3C, CH of triazole), 103.44 (3C, 1-CH), 77.92 (3C, CH sugar), 76.28 (3C, CH sugar), 72.03 (3C, CH sugar), 69.46 (3C, O-*C*H2-Cq), 68.87 (3C, O-*C*H2-CH2-N), 65.40 (3C, O-*C*H2-Cq-NH_2), 62.59 (3C, 6-CH), 61.38 (3C, N=N-C_q-*C*H_2-O), 60.23 (3C, CH sugar), 51.60 (3C, O-CH2-*C*H2-N), 21.47 (3C, OCH3).

Maleimido-TriGlc2NTs (16)

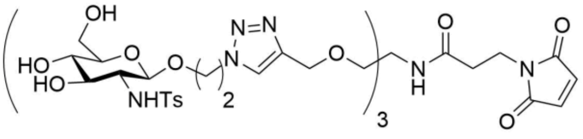

3-Maleinimidopropionic acid (21 µmol, 3.5 mg), HATU (21 µmol, 7.9 mg) and triethylamine (2.8 µmol, 4 µL) were dissolved in 200 µL DMF. After 1 min pre-activation time 126 µL of this reaction mixture was added to a solution of TriGlc2NTs **15** (4.2 µmol, 6 mg, 1 eq) in 5 mL dry DMF. UPLC control showed complete conversion after a few minutes, the solvents were removed in vacuo and the residue purified by preparative HPLC (10 % to 50 % B in A within in 30 min). Analytical HPLC: R_t = 2.7 min (10 - 50 % B in 4 min). ESI-MS: m/z $[M+2H]^{2+}_{calc}$ = 797.3; m/z $[M+2H]^{2+}_{obs}$ = 797.6. ^{1}H NMR (500 MHz, MeOD) δ 7.96 (s, 3H, CH of triazole), 7.68 (d, *J* = 8.2 Hz, 6H, Ph-H), 7.20 (d, *J* = 8.2 Hz, 6H, Ph-H), 6.71 (s, 2H, CH=CH), 4.59 – 4.50 (m, 6H, CH_2-Cq-N=N), 4.40 (ddd, *J* = 14.3, 7.3, 3.4 Hz, 3H, O-CH_2-CH_{2a}-N), 4.30 – 4.22 (m, 6H, O-CH_2-CH_{2b}-N, 1-CH), 3.96 (ddd, *J* = 9.7, 6.0, 3.5 Hz, 3H, O-CH_{2a}-CH_2-N), 3.83 (dd, *J* = 12.0, 2.1 Hz, 3H, 6-CHa), 3.81 (s, 6H, H_2N-Cq-C*H_2*-O), 3.71 – 3.60 (m, 5H, 6-CHb, C_q-CH_2-CH_2-N), 3.45 (ddd, *J* = 7.2, 6.5, 3.4 Hz, 3H, O-CH_{2b}-CH_2-N), 3.35 – 3.14 (m, 12H, 2-CH,3-CH, 4-CH, 5-CH), 2.46 – 2.38 (m, 2H, C_q-CH_2-*CH_2*-N), 2.34 (s, 9H, OCH_3)

7. Synthesis of Mercaptomethylated PNA monomer

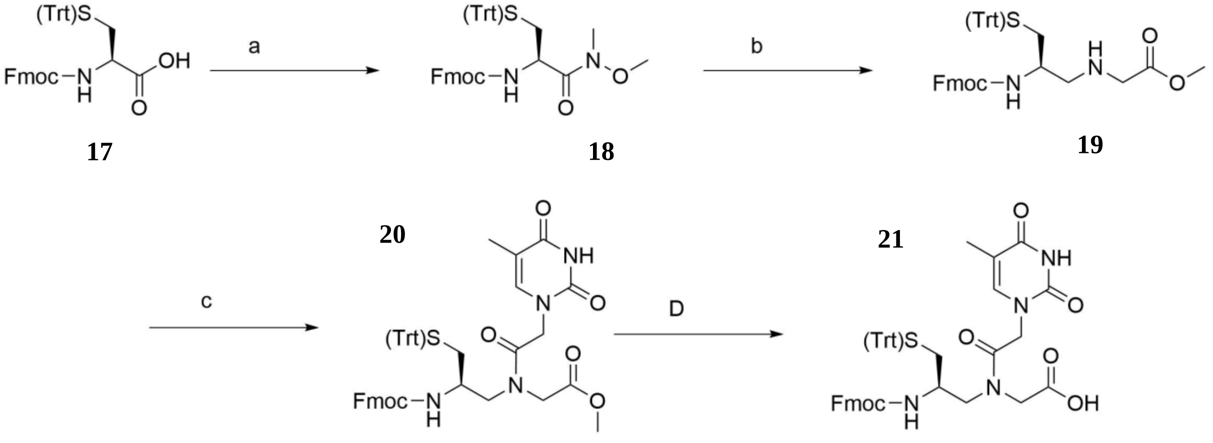

Scheme S3. a) 2.2 eq NMM, 1.1 eq isobutyl chloformate;1 eq N,N-dimethylhydroxylamine hydrochloride, CH_2Cl_2, −10 °C , rt, 17 h, 70 %; b) 1) 2 eq $LiAlH_4$, THF, −72 °C; 1 h; 2) 3 eq glycine methylester hydrochloride; 1 eq $NaCNBH_4$, MeOH, THF; rt; 32 %; c) 1.5 eq thymine acetic acid; 2.25 eq pivaloyl chloride; 6 eq NMM, 1:1 CH_3CN/DMF, −10 °C, rt; 58 %; d) 2 eq LiOH, H_2O, THF, 0 °C, rt, 67 %. Spectroscopic data were in accordance with literature values.[3]

8. Synthesis of Glc2NTs-PNA conjugates

Automated solid-phase PNA synthesis: Linear solid-phase PNA synthesis was performed by using an Intavis ResPep parallel synthesizer and Intavis microscale columns. TentaGel R RAM resin (typical loading: 0.20 mmol/g, 2μmol scale) from Rapp Polymers (Tübingen, Germany) was allowed to swell in DMF for 30 min and then transferred to the synthesizer.
Fmoc cleavage: 250 μL DMF/Piperidine (4:1, v/v) was added to the resin. After 2 min the resin was washed with 300 μL DMF (3x). The cleavage and washing step were repeated.
Coupling of amino acid: 54 μL HCTU (5.4 eq, 0.2 M in NMP), 30 μL NMM (12.0 eq, 0.8 M in NMP) and 40 μL Boc-protected lysine (6.0 eq, 0.3 M in NMP) were mixed in a preactivation vessel. After 2 min, the preactivation solution was transferred onto the resin. After 30 min, the resin was washed with 200 μL DMF (3x) and the coupling was repeated.
Coupling of PNA monomer: 36 μL HCTU (3.6 eq, 0.2 M in NMP), 20 μL NMM (8.0 eq, 0.8 M in NMP) and 40 μL PNA monomer (4.0 eq, 0.2 M in NMP) were mixed in a preactivation vessel. After 2 min, the preactivation solution was transferred onto the resin. After 39 min, the resin was washed with 200 μL DMF (3x) and the coupling was repeated.
Capping: 250 μL DMF/Ac2O/2,6-lutidine (89:5:6, v/v/v) was added to the resin for 2 min. The resin was washed with DMF (300 μL, 5x).
Cleavage from the solid support: Non-thiol-containing PNA oligomers were cleaved by addition of a solution of TFA/H_2O/*i*PrSiH (1 mL, 90:5:5, v/v/v) to the resin, whereas thiol-containing PNA oligomers were cleaved by addition of TFA/*i*PrSiH/EDT (1 mL, 95:3:2, v/v/v) to the resin for 90 min. The suspension was filtered and the resin was washed with TFA (250 μL, 2x). Cold diethyl ether (13.5 mL) was added to the combined filtrates. The turbid mixture was centrifugated for 15 min (4000 rpm, 4 °C). The precipitate was washed with cold diethyl ether (1 mL) and dried under an argon stream.
Purification: The crude product was dissolved in water/acetonitrile (97:3, v/v) and purified by semi-preparative HPLC (3→30 % B in 30 min). Yields for PNA synthesis were between 10 and 30 %.
Sugar-PNA conjugation: One equivalent of mercaptomethylPNA (0.35 – 1.0 mM in water and 2 equivalents of maleimido-Glc2NTs **11** or malimido-TriGlc2NTs **16** (5 – 20 mM in water) were diluted to a final 100 μM PNA concentration in freshly degased sodium dihydrogenphosphate buffer (10 mM, pH 6.6). The reaction mixture was shaken at room temperature and progress of the reaction was monitored by UPLC analysis (3-30% B in 2 min). After complete ligation, the reaction mixture was acidified and lyophilized. The residue was dissolved in water (0.1 % trifluoroacetic acid) and purified by semi-preparative HPLC (3-30 % B in 30 min). Lyophilization afforded the product. **Yields**: 40 – 70 %

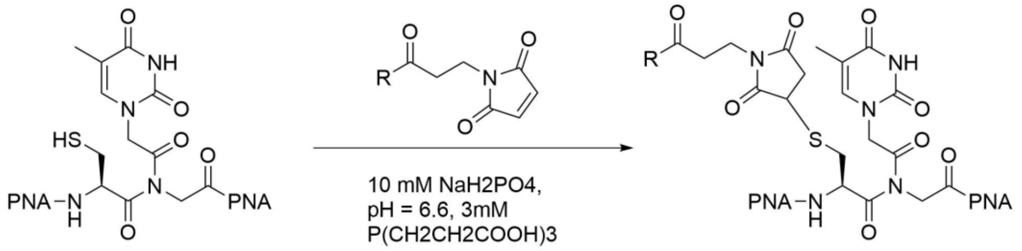

Figure S2. Thiol-maleimide ligation of maleimide-functionalized ligand to a thiolmethylated PNA oligomer (left). R= Glc2NTs (10) or TriGlc2NTs (15)

HPLC traces of PNA oligomers

Unmodified PNA oligmers

P1 **H-DDtcatcgccttctaK-NH$_2$ (P1):** ε_{260} = 116.100 L·mol^{-1}·cm^{-1}, t_R (HPLC): 1.47 min (3 − 30 % B in 2 min). $C_{152}H_{199}N_{71}O_{49}$. ESI-MS: m/z = 952.4 [M+4H]$^{4+}$, calcd.: 952.2), 762.3 [M+5H]$^{5+}$, calcd.: 761.9), 635.2 [M+6H]$^{6+}$, calcd.: 635.1)

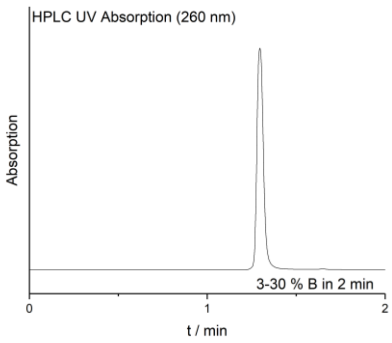

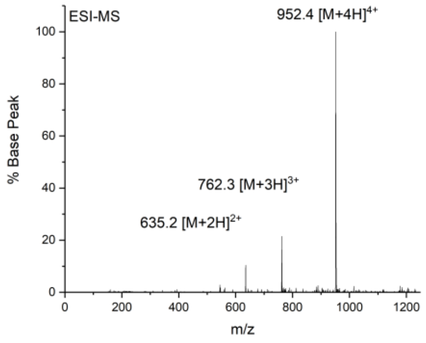

Thiol-modified PNA oligomers

S1 **H-DD acct(~CH$_2$SH)atggactttK-NH$_2$:** ε_{260} = 128.300 L·mol^{-1}·cm^{-1}, t_R (HPLC): 1.49 min (3 − 30 % B in 2 min). $C_{155}H_{201}N_{75}O_{48}S$. ESI-MS: m/z = 979.9 ([M+4H]$^{4+}$, calcd.: 979.5), 784.1 ([M+5H]$^{5+}$, calcd.: 783.9), 653.7 [M+6H]$^{6+}$, calcd.: 653.5)

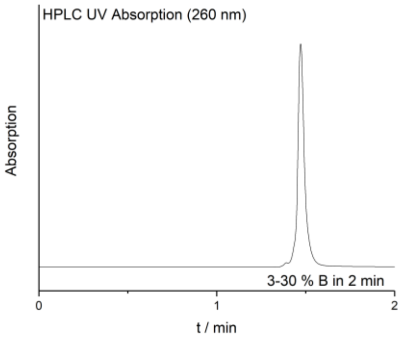

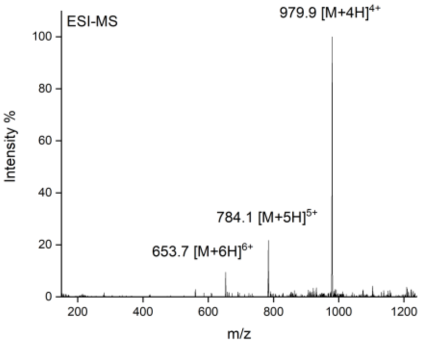

S2 **H-actt(~CH$_2$SH)acttcacgcK-NH$_2$:** ε_{260} = 121.000 L·mol^{-1}·cm^{-1}, tR (HPLC): 1.54 min (3 − 30 % B in 2 min). $C_{145}H_{190}N_{72}O_{41}S$. ESI-MS: m/z = 1210.5 [M+3H]$^{3+}$, calcd.: 1210.9), 907.9 ([M+4H]$^{4+}$, calcd.: 908.4), 726.9 ([M+5H]$^{5+}$, calcd.: 726.9)

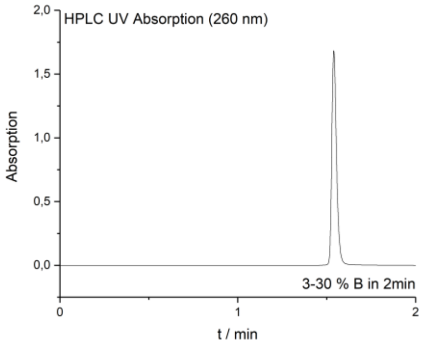

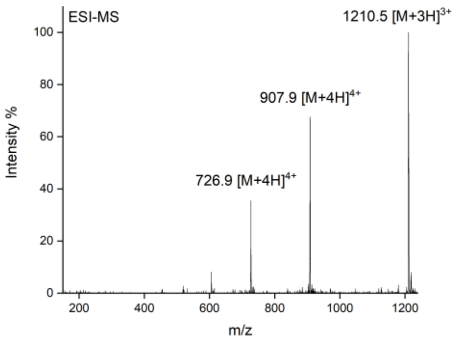

S3 **H-DDatgctacgtt(~CH$_2$SH)gacK-NH$_2$:** ε260 = 131200 L·mol^{-1}·cm^{-1}, tR (HPLC): 1.40 min (3 − 30 % B in 2 min). $C_{155}H_{200}N_{78}O_{47}S$. ESI-MS: m/z = 985.9 ([M+4H]$^{4+}$, calcd.: 985.5), 789.1([M+5H]$^{5+}$, calcd.: 788.9), 657.4([M+6H]$^{6+}$,, calcd.: 657.6),

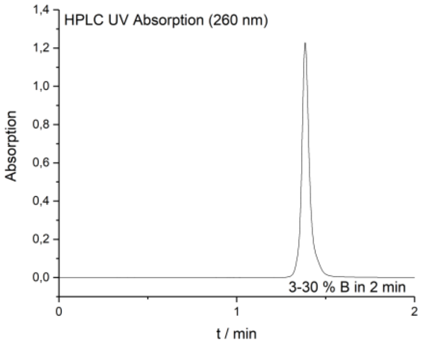

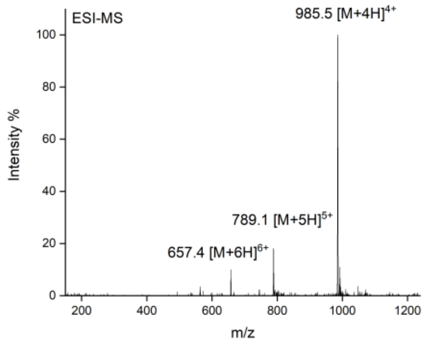

S4 **H- aactcctact(~CH$_2$SH)cct K-NH$_2$:** ε260 = 115900 L·mol^{-1}·cm^{-1}, tR (HPLC): 1.46 min (3 − 30 % B in 2 min). $C_{144}H_{190}N_{70}O_{41}S$. ESI-MS: m/z = 1197.3 ([M+3H]$^{3+}$,calcd.: 1197.5), 897.9 ([M+4H]$^{4+}$,calcd.: 898.4), 718.8([M+5H]$^{5+}$, calcd.: 718.9)

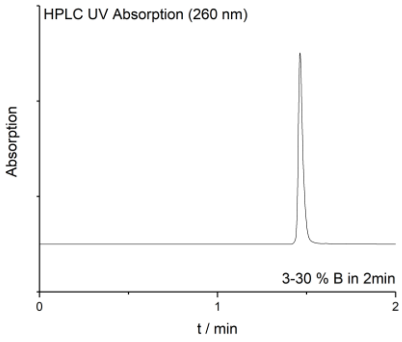

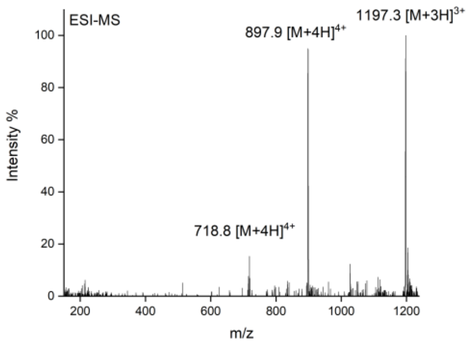

S5 **H-DDatac at(~CH_2SH)cc aacacK -NH_2:** ε_{260} = 132800 L·mol^{-1}·cm^{-1}, tR (HPLC): 1.60 min (3 − 30 % B in 2 min). $C_{154}H_{200}N_{80}O_{42}S$. ESI-MS: m/z = 966.9 ($[M+4H]^{4+}$, calcd.: 965.9), 773.4($[M+5H]^{5+}$, calcd.: 772.9), 644.9($[M+6H]^{6+}$, calcd.: 644.3),

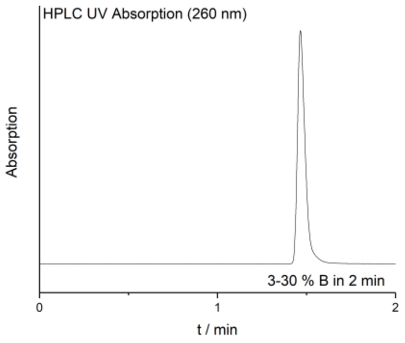

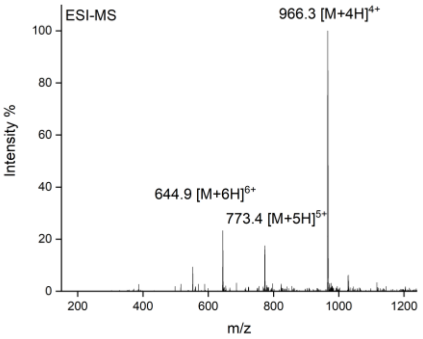

S6 **H-DDtcat(~CH_2SH)tcact(~CH_2SH)cggcK-NH2:** ε_{260} = 119000 L·mol^{-1}·cm^{-1}, tR (HPLC): 1.60 min (3 − 30 % B in 2 min). $C_{154}H_{202}N_{74}O_{48}S_2$. ESI-MS: m/z = 981.5 ($[M+4H]^{4+}$, calcd.: 981.4), 785.5($[M+5H]^{5+}$, calcd.: 785.3), 654.8($[M+6H]^{6+}$, calcd.: 654.6),

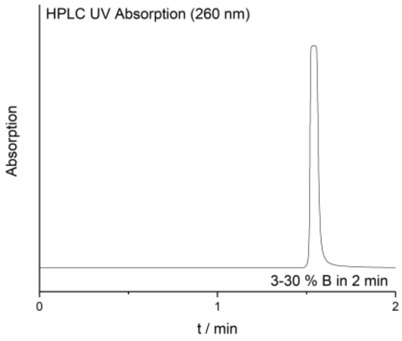

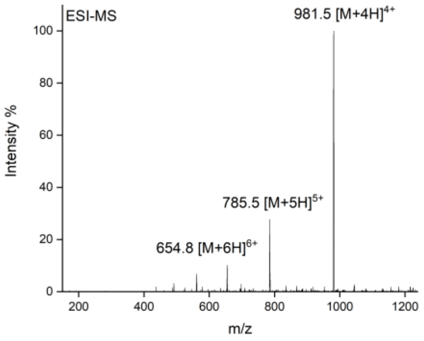

Glc2NTs-PNA conjugates

P2 **H-DD acct(CH_2S~Glc2NTs)atggactt tK-NH_2 (P3):** ε_{260} = 128300 L·mol^{-1}·cm^{-1}; tR (HPLC): 1.69 min (3 − 30 % B in 2 min). $C_{177}H_{230}N_{78}O_{58}S_2$. ESI-MS: m/z = 1111.4 ($[M+4H]^{4+}$, calcd.: 1111.7), 889.3($[M+5H]^{5+}$, calcd.: 889.5), 741.4($[M+6H]^{6+}$, calcd.: 741.5),

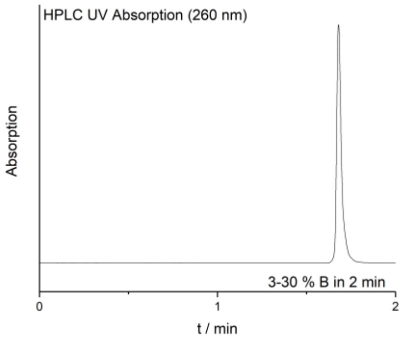

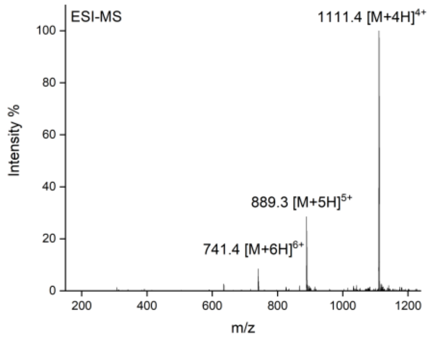

P3 **H- actt(CH_2S~Glc2NTs) acttcacgc K-NH_2:** ε_{260} = 121000 L·mol^{-1}·cm^{-1}, tR (HPLC): 1.72 min (3 − 30 % B in 2 min). $C_{167}H_{219}N_{75}O_{51}S_2$. ESI-MS: m/z = 1040.5 ([M+4H]$^{4+}$, calcd.: 1040.2), 832.6 ([M+5H]$^{5+}$, calcd.: 832.3), 694.0 ([M+6H]$^{6+}$, calcd.: 693.7

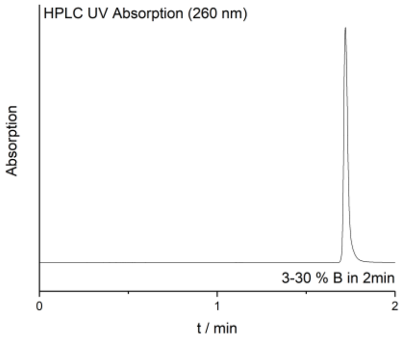

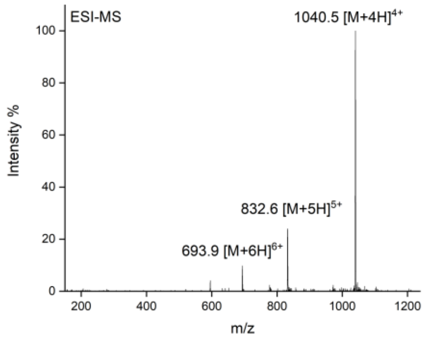

P4 **H-DDatgctacgtt(CH_2S~Glc2NTs)gacK-NH_2 (P5):**: ε_{260} = 131200 L·mol^{-1}·cm^{-1}, tR (HPLC): 1.51 min (3 − 30 % B in 2 min). $C_{178}H_{229}N_{81}O_{57}S_2$. ESI-MS: m/z = 1117.6 ([M+4H]$^{4+}$, calcd.: 1117.9), 894.5([M+5H]$^{5+}$, calcd.: 894.4), 745.2([M+6H]$^{6+}$, calcd.: 745.6),

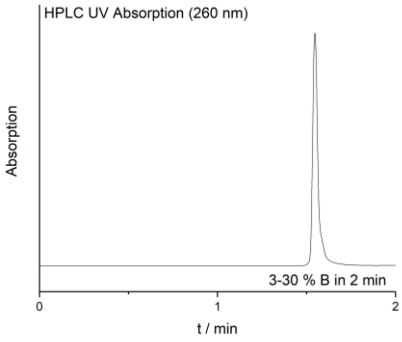

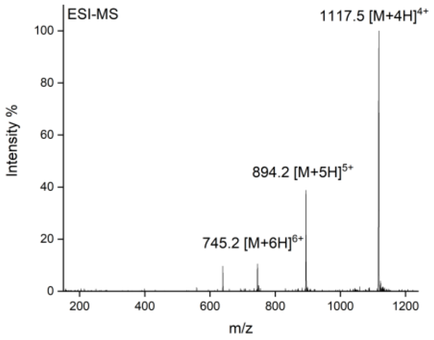

P5 **H-aactcctact(CH_2S~Glc2NTs)cctK-NH_2:** ε_{260} = 115900 L·mol^{-1}·cm^{-1}, tR (HPLC): 1.53 min (3 − 30 % B in 2 min). $C_{166}H_{219}N_{73}O_{51}S_2$. ESI-MS: m/z = 1030.2 ([M+4H]$^{4+}$, calcd.: 1030.2), 824.6([M+5H]$^{5+}$, calcd.: 824.3), 687.3([M+6H]$^{6+}$, calcd.: 687.1),

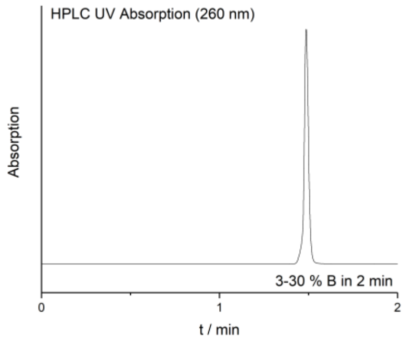

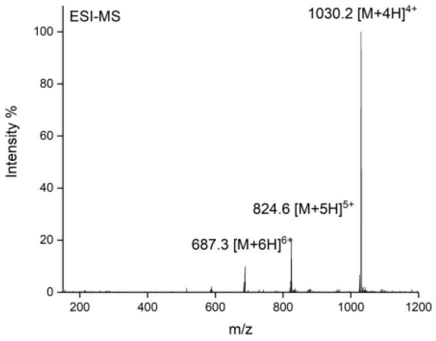

P6 **H-DDatacat(CH2S~Glc2NTs)ccaacac -NH2:** ε_{260} = 132800 L·mol^{-1}·cm^{-1}, tR (HPLC): 1.39 min (3 − 30 % B in 2 min). $C_{154}H_{200}N_{80}O_{42}S$. ESI-MS: m/z = 1098.1 ([M+4H]$^{4+}$, calcd.: 1097.9), 878.7([M+5H]$^{5+}$, calcd.: 878.5), 732.7([M+6H]$^{6+}$, calcd.: 732.3)

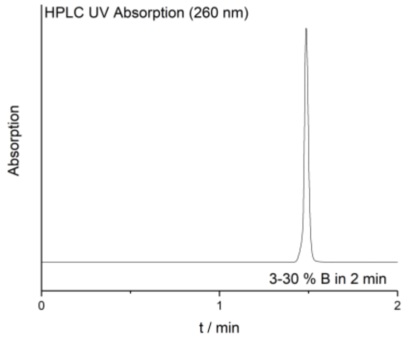

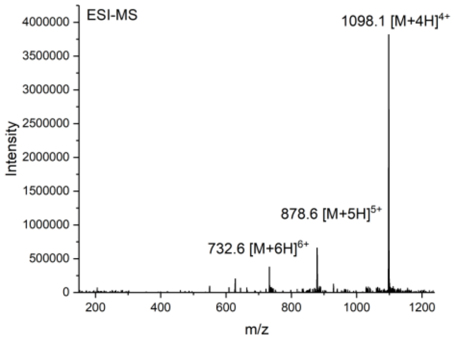

P7 **H-DDtcat(CH2S~Glc2NTs)tcact(CH2S~Glc2NTs)cggcK-NH2:** ε_{260} = 119000 L·mol^{-1}·cm^{-1}, tR (HPLC): 1.77 min (3 − 30 % B in 2 min). $C_{176}H_{231}N_{77}O_{58}S_4$. ESI-MS: m/z = 996.5 ([M+5H]$^{5+}$, calcd.: 996.1), 830.5([M+6H]$^{6+}$, calcd.: 830.3)

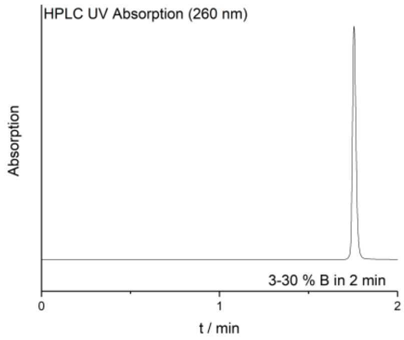

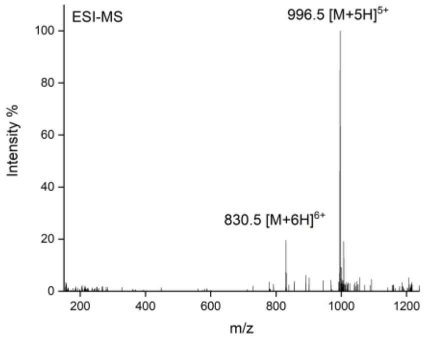

TriGlc2NTs-PNA-conjugates

P8 **H-DDacc t(CH_2S~TriGlc2NTs)atggactttK-NH_2:** ε_{260} = 128300 L·mol^{-1}·cm^{-1}, tR (HPLC): 2.15 min (3 − 30 % B in 4 min). $C_{220}H_{289}N_{89}O_{75}S_4$. ESI-MS: m/z = 1102.6 ([M+5H]$^{5+}$, calcd.: 1102.7), 919.3([M+6H]$^{6+}$, calcd.: 919.1), ([M+7H]$^{7+}$, calcd.: 787.9)

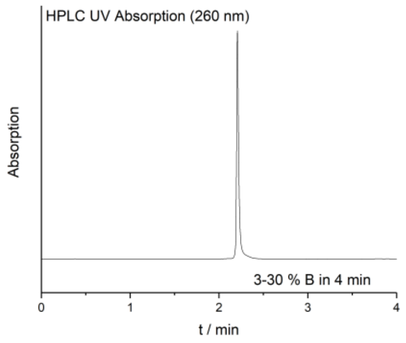

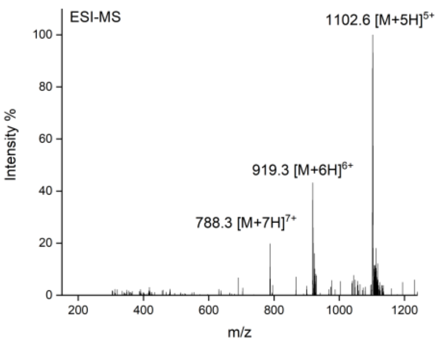

P9 **H-DDatgctacgtt(CH_2S~TriGlc2NTs)gacK-NH_2:** ε_{260} = 131200 L·mol^{-1}·cm^{-1}, tR (HPLC): 2.0 min (3 − 30 % B in 4 min). $C_{220}H_{229}N_{92}O_{74}S_4$. ESI-MS: m/z = 1107.5 ([M+5H]$^{5+}$, calcd.: 1107.7), 923.6([M+6H]$^{6+}$, calcd.: 923.2), 792.6([M+7H]$^{7+}$, calcd.: 791.5

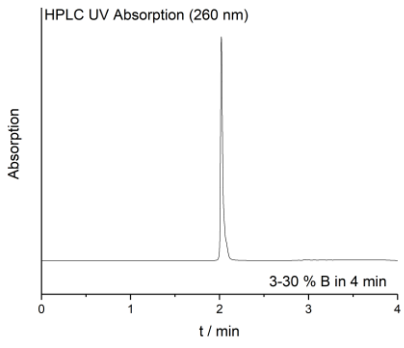

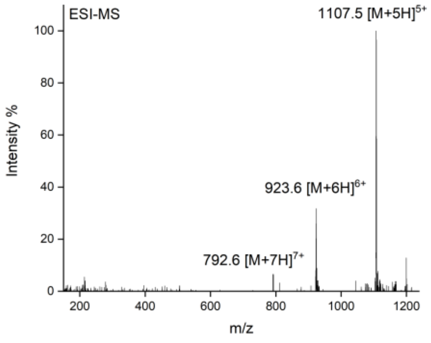

9. Ligand-PNA-DNA complexes

DNA templates

Cy5 - 5'- TAG AAG GCG ATG ATA GAA GGC GAT GAT AGA AGG CGA TGA -3' (**T01**)

5'- AGG AGT AGG AGT TTA GAA GGC GAT GAT AGA AGG CGA TGA -3' (**T02**)

5'- GTC AAC GTA GCA TTA GAA GGC GAT GAT AGA AGG CGA TGA -3' (**T03**)

5'- TAG AAG GCG ATG ATA GAA GGC GAT GAG CCG AGT GAA TGA -3' (**T04**)

5'- TAG AAG GCG ATG AAA AGT CCA TAG GTG TCA ACG TAG CAT -3' (**T05**)

5'- TAG AAG GCG ATG AGT GTT GGA TGT ATG TCA ACG TAG CAT -3' (**T06**)

5'- TAG AAG GCG ATG AGC GTG AAG TAA GTG CGT GAA GTA AGT -3' (**T07**)

5'- TAG AAG GCG ATG AAA AGT CCA TAG GTA AAG TCC ATA GGT -3' (**T08**)

Cy5 - 5'- TAG AAG GCG ATG AAA AGT CCA TAG GTA AAG TCC ATA GGT -3' (**T9**)

Atto647- 5'- TAG AAG GCG ATG AAA AGT CCA TAG GTA AAG TCC ATA GGT -3' (**T10**)

5'- TAG AAG GCG ATG AGT GTT GGA TGT ATA AAG TCC ATA GGT -3' (**T11**)

5'- TAG AAG GCG ATG AAG GAG TAG GAG TTG CGT GAA GTA AGT -3' (**T12**)

5'- AGG AGT AGG AGT TTA GAA GGC GAT GAA GGA GTA GGA GTT -3' (**T13**)

5'- GTC AAC GTA GCA TTA GAA GGC GAT GAG TCA ACG TAG CAT -3' (**T14**)

5'- GTC AAC GTA GCA TTA GAA GGC GAT GAA AAG TCC ATA GGT -3' (**T15**)

Ligand-PNA-DNA duplex composition and T_M

Formation of Ligand-PNA-DNA complexes: To a DNA template (T01 – T15) appropriate equivalents of a PNA oligomers (P1 – P9) were added. The solution was lyophilized to a white foam and buffer added for the desired concentration. The mixture was heated to 80°C in a thermo shaker for 5 min and slowly left to cool down to r.t. to allow for hybridization.

Table S1 Composition of the Glc2NTs-PNA•DNA complexes. [a] Number of nucleotides between the ligands + 1. [b] Estimated distance between the ligands based on 3.25 Å average rise per base pair in a DNA·PNA duplex

Complex		DNA template	nt [a]	d/Å [b]	PNAs	T_M / °C
Free-Ligand-Complex-Cy5	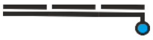	T01	-	-	3xP1	63
Mono-Glc2NTs	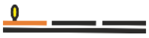	T02	-	-	2xP1; P5	62
Mono-Glc2NTs	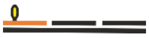	T03	-	-	2xP1; P4	62
Biv-Glc2NTs-5	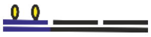	T04	5	16	2xP1; P7	64
Biv-Glc2NTs-7	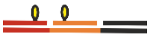	T05	7	23	P1; P2; P4	66
Biv-Glc2NTs-9	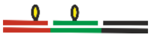	T06	9	29	P1; P4; P6	63
Biv-Glc2NTs-13	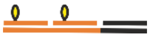	T07	13	42	P1; 2xP3	64
Biv-Glc2NTs-13	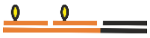	TO8	13	42	P1, 2xP2	64
Biv-Glc2NTs-13-Cy5	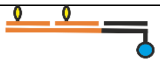	T09	13	42	P1, 2xP2	-
Biv-Glc2NTs-13-Atto647	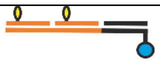	T10	13	42	P1, 2xP2	-
Biv-Glc2NTs-15	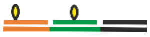	T11	15	49	P1, P2, P6	62

Biv-Glc2NTs-19	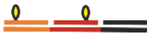	T12	19	62	P1; P3; P5	62
Biv-Glc2NTs-26	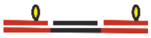	T13	26	84	P1; 2xP5	62
Biv-Glc2NTs-26	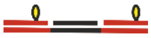	T14	26	84	P1; 2xP4	68
Biv-Glc2NTs-32	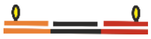	T15	32	104	P1; P2; P4	66

Table S2 Composition of the TriGlc2NTs-PNA•DNA complexes [a] Number of nucleotides between the ligands + 1. [b] Estimated distance between the ligands based on 3.25 Å average rise per base pair in a DNA·PNA duplex.

Complex		template	nt [a]	d/Å[b]	PNAs	T_M / °C
Mono-TriGlc2NTs	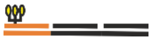	T03	-	-	2xP1; P9	65
Biv-TriGlc2NTs-7	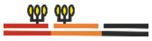	T05	7	23	P1; P8; P9	66
Biv-TriGlc2NTs-13	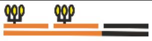	T08	13	42	P1; 2XP8	64
Biv-TriGlc2NTs-13-Cy5	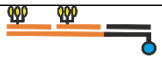	T09	13	42	P1; 2XP8	-
Biv-TriGlc2NTs-26	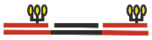	T14	26	84	P1; 2xP9	68
Biv-TriGlc2NTs-32	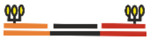	T15	32	104	P1; P8; P9	66

Melting curve

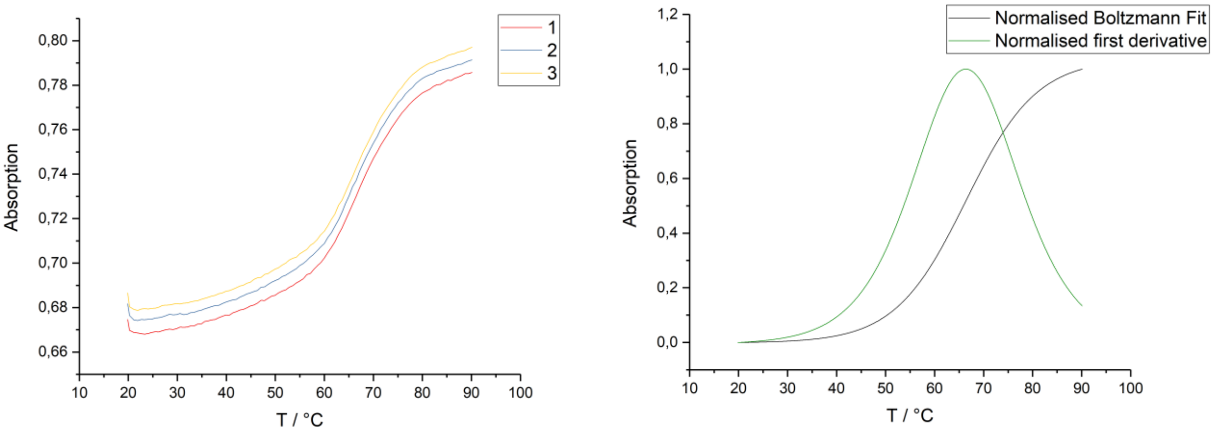

Figure S3. Exemplary UV melting curves (red, blue, yellow), normalized Boltzmann Fit (black) and normalized first derivative(green) for Biv-TriGlc2NTs-32. The absorbance at 260 nm was monitored during a thermal cycle (3 cycles from 20 – 90 °C in 0.5 °C/min), the curves inflexion points were calculated and averaged over all cycles. Conditions: 1 µM complex, 10 mM NaH_2PO_4, 100 mM NaCl, pH 7.0.

10. Langerin ECD and CRD Receptor Expression and Purification

Langerin extracellular domain. Expression and purification were conducted as previously published.[9] Briefly, the trimeric Langerin extracellular domain (ECD) was expressed insolubly in *E. coli* BL21* (DE3) (Invitrogen). Following enzymatic cell lysis, inclusion bodies were harvested and subsequently solubilized. The sample was centrifuged and the Langerin ECD was refolded overnight via rapid dilution. Next, the sample was dialyzed overnight, centrifuged and purified via mannan-agarose affinity chromatography (Sigma Aldrich). For ^{19}F R2-filtered NMR experiments, the buffer was exchanged to 25 mM Tris, 150 mM NaCl, 5 mM $CaCl_2$, pH 7.8. For SPR experiments, the buffer was exchanged to 20 mM HEPES, 150 mM NaCl, 1 mM $CaCl_2$, pH 7.4. The concentration of Langerin ECD was determined via UV spectroscopy (ε_{280} = 56.170 mol^{-1} cm^{-1}). Purity and monodispersity of Langerin ECD samples were analyzed via SDS PAGE and DLS.

Langerin carbohydrate recognition domain. Expression and purification were conducted as previously published.[9] Briefly, the monomeric ^{15}N-labeled Langerin carbohydrate recognition domain (CRD) was expressed insolubly in *E. coli* BL21* (DE3) (Invitrogen). Following enzymatic cell lysis, inclusion bodies were harvested and subsequently solubilized. The sample was centrifuged and the Langerin CRDs were refolded overnight via rapid dilution. Next, the sample was dialyzed overnight, centrifuged and purified via StrepTactin affinity chromatography (Iba). After an additional dialysis step overnight, the sample was centrifuged and the buffer was exchanged to 25 mM HEPES, 150 mM NaCl , pH 7.0. The concentration of Langerin CRDs was determined via UV spectroscopy (ε_{280} = 56.170 mol^{-1} cm^{-1}). Purity and monodispersity of Langerin CRD samples were analyzed via SDS PAGE and DLS.

11. ^{19}F-NMR Assay Inhibition Experiments

The ^{19}F-NMR Assay has been previously described by Wamhoff et al.[10] Langerin ECD and CRD were obtained as described above.

Experiments with the Langerin ECD were performed at a receptor concentration of 50 µM or 25 µM in 25 mM Tris with 10 % DMSO, 10% D_2O, 150 mM NaCl and 5 mM $CaCl_2$ at pH 7.8 and 25° C.
Experiments with the Langerin CRD were performed at a receptor concentration of 50 µM in 25 mM HEPES with 10% DMSO, 10% D_2O, 150 mM NaCl and 5 mM $CaCl_2$ at pH 7.0 and 25°C.
TFA served as an internal reference at a concentration of 50 µM. Apparent relaxation rates R2,obs for the reporter ligand were determined using the CPMG pulse sequence as previously published. [10]

IC_{50} Determination

Remarks: Measurements of IC_{50} values via 19F-NMR were considered as am initial screen for which hits were subsequently validated using an orthogonal assay (see chapter 12). Due to the substantial amount of ligand and protein required for the NMR assay, experiments were conducted as one independent 5-point titration per construct and standard errors of the mean (SEM) for IC_{50} and Hill factor were obtained directly from the fitting procedure. The following SPR experiments were conducted as duplicates We prioritised the value of two orthogonal methods, which we believe gives more reliable results than just one method with extensive

replicates. One could argue that this approach is more robust as it accounts, to some degree, for systematic errors and assay-dependent artefacts Further, the distance affinity screening measurements are not biological experiments, where a large statistical variance must be assumed, but rather physicochemical effects that display lower variance.

IC_{50} values were determined in competitive binding experiments *via* the detection of binding of 0.1 mM ^{19}F-marked reporter ligand (propargyl-2-deoxy-2-trifluoroacetamido-α-mannoside) to either the Langerin ECD or CRD at six competitor concentrations as previously published.[10] Samples were prepared *via* serial dilution. Equation 1 served to derive IC_{50} values and Hills factors p from R2,obs values in a two parameter fit. R2,max represents the relaxation rate at 0.1 mM reporter ligand in presence of receptor and in absence of competitor. The measurements were done in single experiments (five point titration); standard errors were derived directly from the fitting procedures.

Equation 1: IC_{50} $R2,=R2,f+(R2,max-R2,f1+(IC50[I]T)p)$

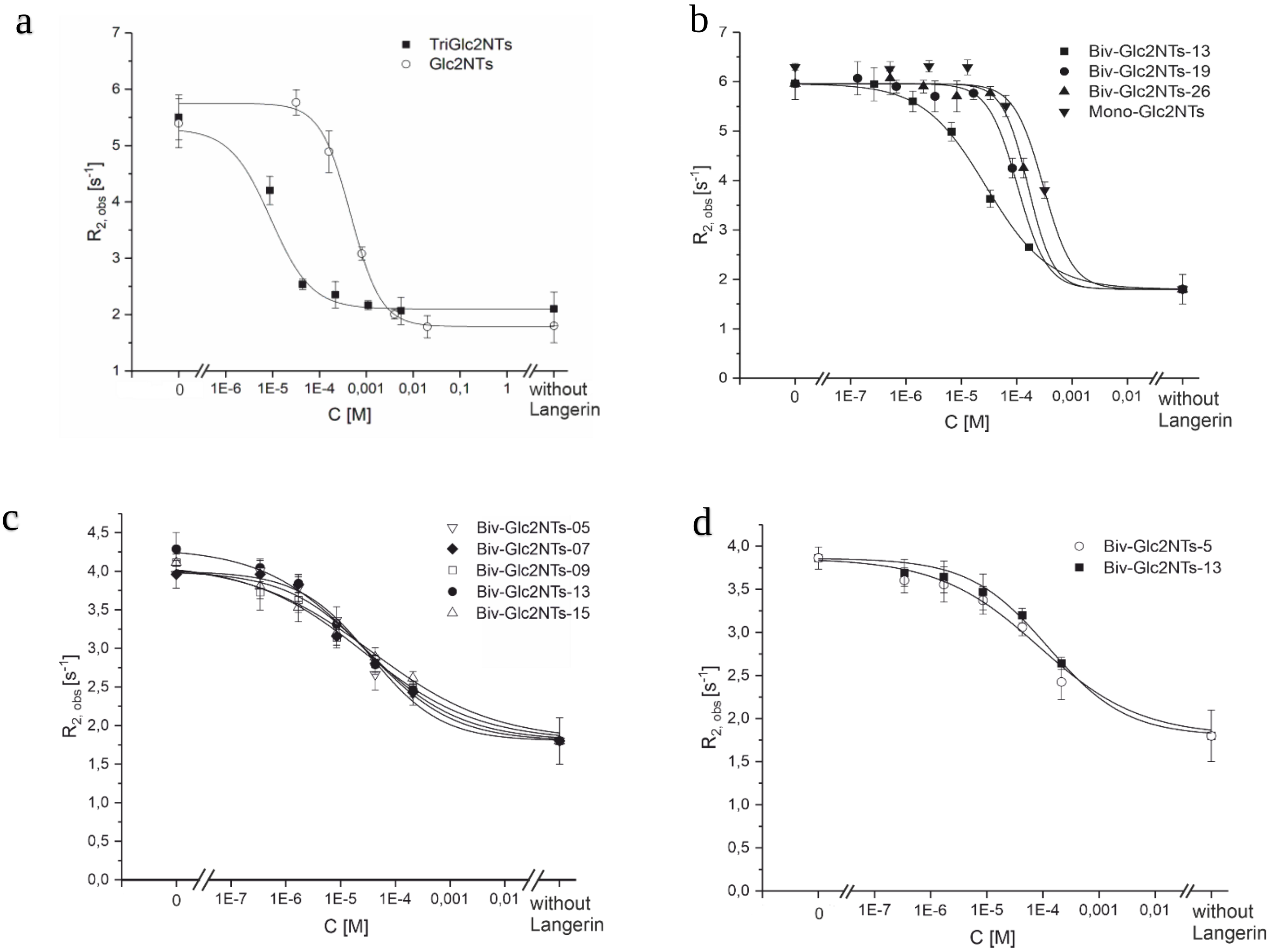

Figure S4. ^{19}F-NMR Assay Inhibition Curves for titrations of Langerin ECD and reporter ligand with A) Glc2NTs and TriGlc2NTs, B) Mono-Glc2NTs, Biv-Glc2NTs-13, Biv-Glc2NTs-19, Biv-Glc2NTs-26 C) Biv-Glc2NTs-05, Biv-Glc2NTs-07, Biv-Glc2NTs-09, Biv-Glc2NTs-13, Biv-Glc2NTs-15. D) Concentration dependent inhibition of the Langerin CRD (50 µM) by Glc2NTs-PNA-DNA duplexes: Biv-Glc2NTs-05, Biv-Glc2NTs-13. Conditions: A), B) 50 µM Langerin ECD, 100 µM reporter ligand. C) 25 µM Langerin ECD, 100 µM reporter ligand. The measurements were done as single experiments; standard errors were derived directly from the fitting procedures.

The error-weighted, non-linear fitting procedure on experimentally determined transversal NMR relaxation constants (R2,obs, which themselves have associated statistical variance and SEMs) has been previously described and validated.1 Consequently, the magnitude of the SEM depends largely on the "goodness of fit" of the R2,obs values, i.e. how well the data points adhere to the assumed Hill equation. Variance stems from two factors: pipetting errors and technical errors from determining R2,obs both potentially resulting in "outliers". We assume the latter to be the main contributing factor as the SNR in some of the 19F NMR spectra when determining fast R2,obs values of more than 5 Hz will be low. This limitation is aggravated by inconsistent automated shimming when using 150 µl NMR sample tubes. Both the low SNR and choice of sample tube are compromises to lower material requirements and increase throughput as previously described.1

In summary, the plotted SEMs reflect the technical error within each data set and the statistical variance in SEMs are due the presence of R2,obs outliers within some of these data sets. These SEMs only give limited insight into the variance when obtaining replicates from independent titration experiments. We fully expect error bars to be consistent under those conditions and have previously demonstrated that KI values can be reliably estimated (σ ≈ 20%) even from single-point experiments.1 We further note that the relative errors obtain biv-Glc2NTs-13 are, in fact, comparable (ECD = 0.09 and CRD = 0.17).

Table S3. IC_{50} values for Glc2NTs-PNA-DNA complexes to the Langerin ECD assessed by a ^{19}F NMR assay

Structure	Compound	Distance[a]	Langerin	IC_{50} / µM	Rel. potency based on Glc2NTs = β-value (valency corrected β-value)
	Glc2NTs	-	ECD[b]	361 ± 28	1 (1)
	TriGlc2NTs	-	ECD[b]	12 ± 2	30 ± 7 (10 ± 2.4)
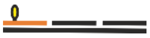	Mono-Glc2NTs	-	ECD[b]	273 ± 32	1.3 ± 0.26 (1.3 ± 0.26)
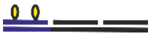	Biv-Glc2NTs-05	16 Å	ECD[c]	35 ± 8	10.3 ± 3.2 (5.2 ± 1.6)
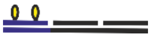	Biv-Glc2NTs-05	16 Å	CRD[b]	60 ± 20	6.0 ± 2.4 (3.0 ± 1.2)
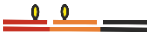	Biv-Glc2NTs-07	23 Å	ECD[c]	37 ± 9	9.7 ± 3.1 (4.9 ±1.6)
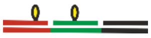	Biv-Glc2NTs-09	29 Å	ECD[c]	25 ± 3	14.4 ± 2.8 (7.2 ± 1.4)
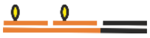	Biv-Glc2NTs-13	42 Å	ECD[b]	25 ± 1	14.4 ± 1.68 (7.2 ± 0.8)
			ECD[c]	23 ± 2	15.7 ± 2.5 (7.8 ± 1.4)
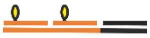	Biv-Glc2NTs-13	42 Å	CRD[b]	120 ± 20	3.0 ± 0.7 (1.5 ± 0.4)
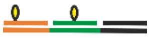	Biv-Glc2NTs-15	49 Å	ECD[c]	36 ± 4	10.0 ± 1.9 (5.0 ± 0.9)
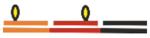	Biv-Glc2NTs-19	62 Å	ECD[b]	126 ± 22	2.9 ± 0.7 (1.4 ± 0.36)
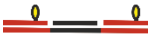	Biv-Glc2NTs-26	84 Å	ECD[b]	198 ± 42	1.8 ± 0.5 (0.9 ± 0.3)

[a] estimates based on 3.25 Å average rise per base pair in a DNA·PNA duplex; [b] 50 µM Langerin; [c] 25 µM Langerin.

Comments: Constructs Biv-Glc2NTs-19 and 26 seem to display steeper curves. However, because they are weaker binders only the highest concentration results inhibition. Therefore, the curve shapes are poorly defined and the hill factors for these constructs were fixed to 1 to allow for a conservative IC_{50} estimation. We express caution and rather not over-interpret the curve shapes. The reason we refrained from using higher concentrations were concerns around solubility of the PNA-DNA constructs. Importantly, the solubility issue was addressed successfully by leveraging statistical rebinding effects (see chapter 12).

With the exception of constructs Biv-Glc2NTs-19 and 26, all other bivalent constructs display reduced p values around 0.5. It should be noted that the ^{19}F-NMR assay was performed at the assay limit, which will affect the hill slopes. The decrease in Hill factors could also be due to an autoinhibitory effect at higher concentrations where two or more individual bivalent constructs bind to a single ECD trimer and compete with chelate binding.
Importantly, data obtained in the SPR assay (except for Biv-TriGlc2NTs-32 and Biv-Glc2NTs-07) displayed hill slopes >1 (chapter 12). This assay was not performed at the assay limit and high concentrations of proteins are avoided. The hill slopes > 1 are indicative of optimal chelate binding.

12. SPR Assay Inhibition Experiments

Experiments were performed on a Biacore X100 instrument (GE Healthcare Europe GmbH Uppsala, Sweden) at 25 °C. Langerin ECD was obtained as described above.[1] The procedure was based on a previously described method.[7] For immobilization of biotinylated α-D-mannose–PAA, the HBS-EP buffer (10 mM HEPES, 150 mM NaCl, 3 mM EDTA, pH 7.4 and 0.005% surfactant P 20) from GE Healthcare Europe GmbH was used. For better performance, the sensor chip was initially conditioned with three consecutive 1 min injections of 1 M NaCl in 50 mM NaOH before starting immobilization. Biotinylated α-D-mannose–PAA (20 mol %; Lectinity Holdings, Moscow, Russia) was immobilized on the measuring channel of a streptavidin coated sensor chip (Sensor Chip SA, GE Healthcare Europe GmbH). On the reference channel of the same sensor chip, biotinylated α-D-galactose–PAA (Lectinity, Moscow, Russia) was immobilized. Running buffer during the assays was 20 mM HEPES, 150 mM NaCl, 1mM $CaCl_2$, pH 7.4, (all Carl Roth GmbH & Co. KG, except $CaCl_2$ from Sigma-Aldrich Chemical Co.). For testing the experimental set up, single cycle kinetics were performed at five concentrations of Langerin, ranging from 62 nM to 5000 nM, and finally a K_D value of 1.5 µM was determined. This study also confirmed the used protein contact times of 120 s, the dissociation time of 300 s and a flow rate of 20 µl/min. The chip surfaces were regenerated at 30 µl/min with 10 mM EDTA pH 8 and a contact time of 60 s.
In the **dose response experiment** before injection, each protein sample ([Langerin]=500 nM) and a serial dilution of the complexes (dissolved in 20 mM HEPES, 150 mM NaCl, 1mM $CaCl_2$, pH 7.4) were incubated for a minimum of 5 min at rt. The samples were injected over the reference and measuring channel. For evaluation the reference channel data were subtracted from the measuring channel data. The Langerin control was measured before and after every dose response series. By calculating the regression between both values an individual Langerin baseline drift was calculated for every dose response measurement. Corrected response values were calculated by dividing the RU of the dose response experiment by the individual Langerin baseline RU. The corrected response values were used for curve creation and IC_{50} fitting procedure. Responses of the sample injections were extracted between report points set at the start of the injection (0 s) and at the end of the dissociation phase (250 s). Each data point represents the mean value (SEM) of 2 measurements (duplicates).
In the **single concentration experiment** the protein sample [Langerin]=500 nM) and the [complexes] = 10 µM (dissolved in 20 mM HEPES, 150 mM NaCl, 1mM $CaCl_2$, pH 7.4) were incubated for a minimum of 5 min at rt. The samples were injected over the reference and measuring channel. For evaluation the reference channel data were subtracted from the measuring channel data.

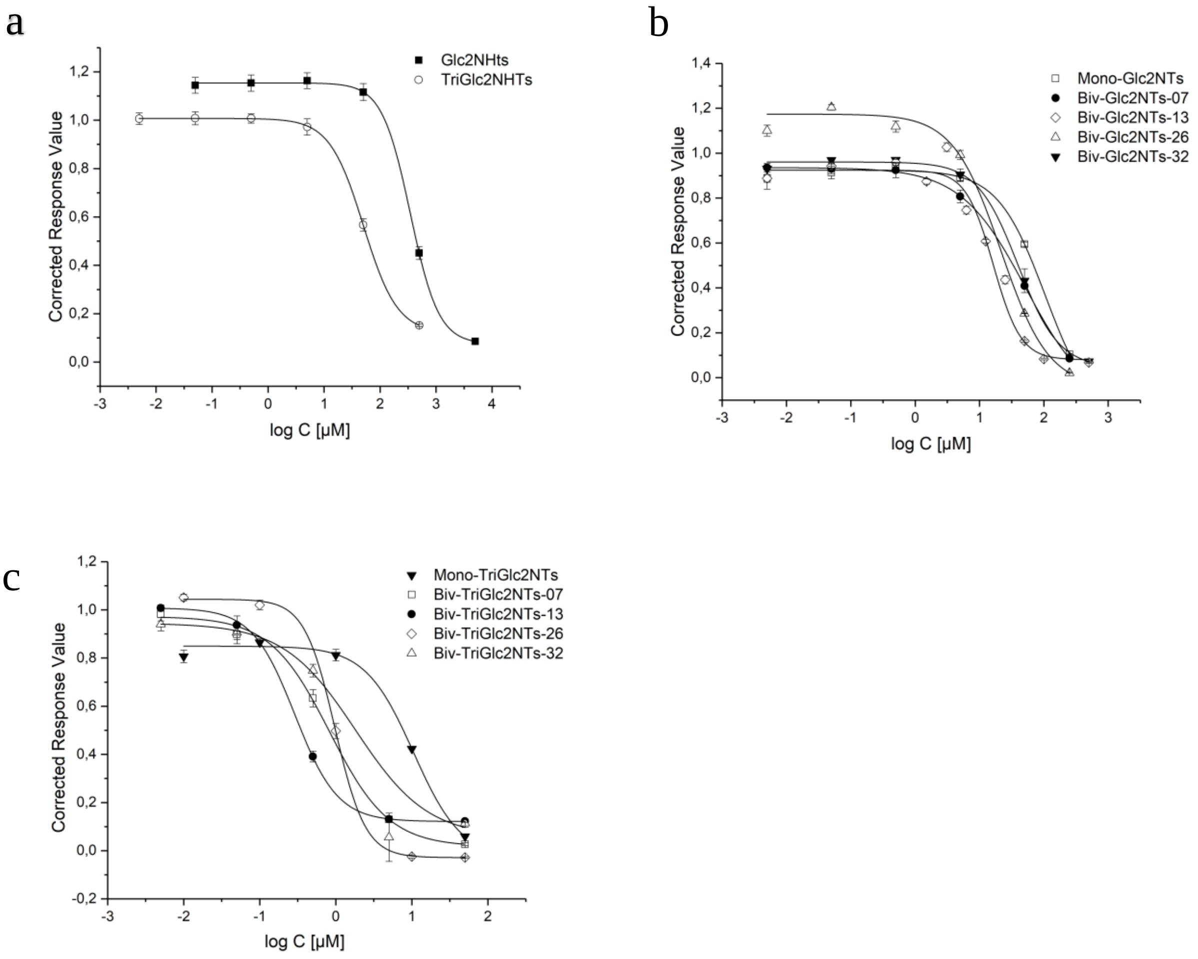

Figure S5. Normalized titration curves determined by SPR. Concentration dependent inhibition of the Langerin ECD by a) the mono ligands Glc2NTs and TriGlc2NTs; b) Glc2NTs-PNA-DNA duplexes (Mono, 07, 13, 26, 32); c) TriGlc2NTs-PNA-DNA duplexes (Mono, 07, 13, 26, 32).

Table S4 SPR signals detected at ligand concentration = 10 µM in three independent replicates.

**Replicate 1**	**Mono-Glc2NTs**	**Biv-Glc2NTs-07**	**Biv-Glc2NTs-13**	**Biv-Glc2NTs-26**	**Biv-Glc2NTs-32**
1	104,7	86,4	77,6	94,8	99,5
2	90,85	81,25	62,1	83,1	83,35
3	93	80,95	65,35	86,05	84,05

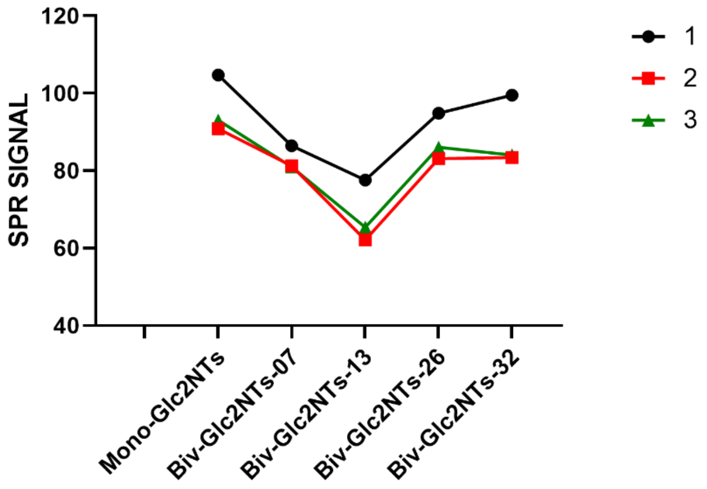

Figure S6 SPR signals detected at ligand concentration = 10 μM in three independent replicates. Conditions: Before injection, the protein sample (500 nM) and a serial dilution of the complexes were incubated for 5 min at rt in 25 mM HEPES buffer with 150 mM NaCl and 5 mM $CaCl_2$ at pH 7.8 and 25° C. The samples were injected over a reference, and α-D-mannose–polyacrylamide chip and the residual Langerin binding measured.

An Anova test was performed with GraphPad to evaluate whether the differences are statistically significant. Experimental design: Each row represents repeated measures; Anova (Gaussian distribution), Geisser-Greenhouse correction. The One-way Anova rejected the null-hypothesis (P<0.05).

Repeated measures ANOVA summary	
Assume sphericity?	No
F	53,45
P value	0,0118
P value summary	*
Statistically significant (P < 0.05)?	Yes
Geisser-Greenhouse's epsilon	0,2902
R square	0,9639
Was the matching effective?	
F	37,65
P value	<0,0001
P value summary	****
Is there significant matching (P < 0.05)?	Yes
R square	0,2534

Additionally, the mean of each column was compared with the mean of column Biv-Glc2NTs-13.

Holm-Sidak's multiple comparisons test	Mean Diff,	Significant?	Summary	Adjusted P Value
Biv-Glc2NHTs-13 vs. Mono-Glc2NTs	-27,83	Yes	**	0,0012
Biv-Glc2NHTs-13 vs. Biv-Glc2NTs-07	-14,52	Yes	*	0,0411
Biv-Glc2NHTs-13 vs. Biv-Glc2NHTs-26	-19,63	Yes	**	0,0077
Biv-Glc2NHTs-13 vs. Biv-Glc2NHTs-32	-20,62	Yes	**	0,0067

Table S5. Binding affinities of Glc2NTs, TriGlc2NTs and the corresponding bivalent ligand-PNA-DNA complexes determined by SPR. Comparison of the β-value (IC$_{50}$(mono)/IC$_{50}$(multi)**)** and valency corrected β-value = β/n, where n corresponds to either the number of Glc2NTs or TriGlc2NTs moieties. Conditions: Before injection, the protein sample (500 nM) and a serial dilution of the complexes were incubated for 5 min at rt in 25 mM HEPES buffer with 150 mM NaCl and 5 mM $CaCl_2$ at pH 7.8 and 25° C. The samples were injected over a reference, and α-D-mannose–polyacrylamide chip and the residual Langerin binding measured. The corrected values were plotted against the concentrations to determine the IC$_{50}$ values.

**Structure**	**Compound**	**Distance[a]**	**IC$_{50}$ /µM**	**β-value based on Glc2NTs (β / n) n = no. of Glc2NHTs**	**β-value based on TriGlc2NTs (β / n) n = no. of TriGlc2NHTs**
	Glc2NTs	-	347 ± 11	1 (1)	-
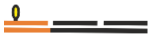	Mono-Glc2NTs	-	105 ± 44	3.3 ± 1.5 (3.3 ± 1.5)	-
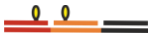	Biv-Glc2NTs-07	23 Å	52 ± 2	6.7 ± 0.5 (3.3 ± 1.4)	-
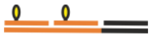	Biv-Glc2NTs-13	42 Å	16 ± 1	21.7 ± 2.0 (10.8 ± 1.0)	-
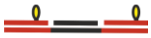	Biv-Glc2NTs-26	84 Å	21 ± 2	16.5 ± 2.1 (8.3 ± 1.1)	-
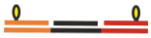	Biv-Glc2NTs-32	104 Å	40 ± 14	8.6 ± 3.3 (4.3 ± 1.7)	-
	TriGlc2NTs	-	50.5 ± 0.5	6.8 ± 0.3 (2.3 ± 0.1)	1 (1)
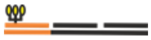	Mono-TriGlc2NTs	-	10,5 ± 2,2	33 ± 8.0 (11 ± 2.7)	4.8 ± 1.1 (4.8 ± 1.1)
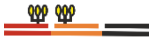	Biv-TriGlc2NTs-07	23 Å	0,8 ± 0,1	433.8 ± 68.1 (72.3 ±11.4)	63.1 ± 8.5 (31.6 ± 4.3)
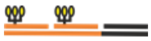	Biv-TriGlc2NTs-13	42 Å	0,3 ± 0,02	1156.7 ± 114.1 (192.7 ± 19.0)	168.3 ± 12.9 (84.2 ± 6.5)
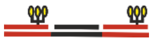	Biv-TriGlc2NTs-26	84 Å	1,0 ± 0,1	347.0 ± 45.8 (57.8 ± 7.6)	50.5 ±5.6 (25.3 ± 2.8)
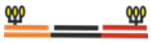	Biv-TriGlc2NTs-32	104 Å	1,8 ± 0,7	192.7 ± 0.42 (32.2 ± 13.5)	28.1 ± 11.2 (14.0 ± 6.0)

[a] estimates based on 3.25 Å average rise per base pair in a DNA·PNA duplex

13. C-type Lectin+ Model Cells

Cell Culture

Raji cells (ATCC) were cultured in RPMI1640 medium (Sigma Aldrich) containing 10% FCS (Biochrom), 100 U*mL^{-1} penicillin and streptomycin (Life Technologies) and GlutaMax-I (Life Technologies). COS-7 cells (ATCC) were cultured in DMEM supplemented with 10% FCS (Pan-Biotech). All cell lines were maintained at 5% CO2 and 37°C.

Establishment of C-Type Lectin+ Model Cells

The production of the model cells has been previously described.[4]

14. Flow Cytometry Assay

Raji cells, suspended in cell culture medium (RPMI1640 (Sigma Aldrich)), were counted, centrifuged at 500 g for 3 min, aspirated, incubated with blocking buffer (0.2 mg/mL Salmon Sperm DNA, 0.2 % BSA in PBS) centrifuged at 500 g for 3 min, aspirated resuspended in culture medium at 37° C and 5% CO2. 50,000 cells were added to the 96 well microtiter plates (Nunc) to obtain a volume of 100 µl. To monitor internalization and binding, Biv-Glc2NTs-12 or Biv-TriGlc2NTs-13 in HBS (20 mM HEPES, 150 mM NaCl, 1mM $CaCl_2$, pH 7.4) were added to the cells at a final concentration of either 660 nM (Method I) or 66 nM (Method II). In case of control experiment 250 µg·mL^{-1} mannan in PBS was added to the cells and constructs. The cells and constructs were incubated for 45 min at 4° C and subsequently centrifuged at 500 g for 3 min. Cells were aspirated and resuspended in cell media for 60 min at 37° C and 5% CO2. Afterwards, the cells were resuspended in fresh cell culture medium before analyzing (Method I) or directly analysed without fresh medium (Method II). Internalization and binding of fluorophore marked complexes was evaluated by flow cytometry on an BA Accuri C6 Plus Flow Cytometer equipped with an autosampler by detecting the conjugated dye Cyanine5 with a 640 nm laser and > 670 filter set. 5000 events were measured for every well. The data was analysed with CFlow Plus. For normalization the Mean Fluorescent Intensity (MFI) of the Raji cells (autofluorescence) was subtracted from the measured MFI of cells with ligands.

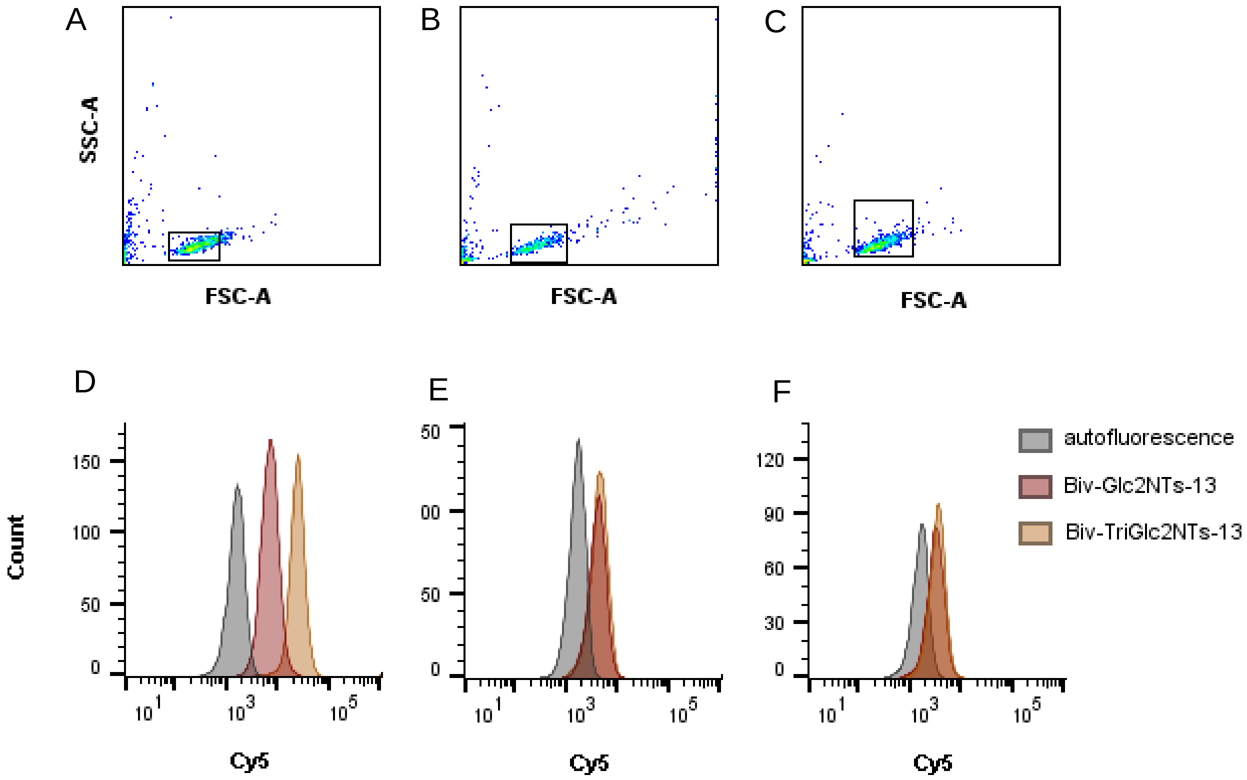

Figure S7. Example of gating strategy A)Langerin$^+$, B) wild type, C) DC-SIGN$^+$ Raji cells; Example of histograms Raji cells (grey), Raji cells + Biv-Glc2NTs-13-Cy5 (red), and Raji cells + Biv-TriGlc2NTs-13-Cy5 (orange) D) Langerin$^+$, E) wild type, F) DC-SIGN$^+$.(Method I)

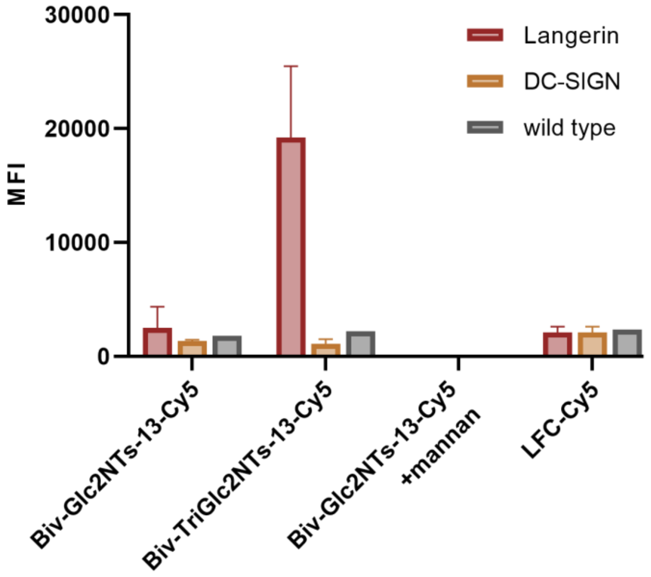

Figure S8. Internalization experiment at 66 nM ligand concentration (Method II)

15. Confocal Microscopy of COS-7 cells

COS-7 cells were seeded on glass-bottom culture dish (Miltenyi Biotec) with 10 % FBS supplemented DMEM (Dulbecco's Modified Eagle's Medium) and incubated in cell culture incubator (Thermo) for overnight. Then the cell media was mixed with the 70 nM of Biv-Glc2NTs-13-Atto647 and the cells were incubated for 10 min. The supernatant was replaced with 37 °C PBS three times and the dish was placed in a live-cell imaging chamber equipped on SP8 inverted confocal microscope (Leica).

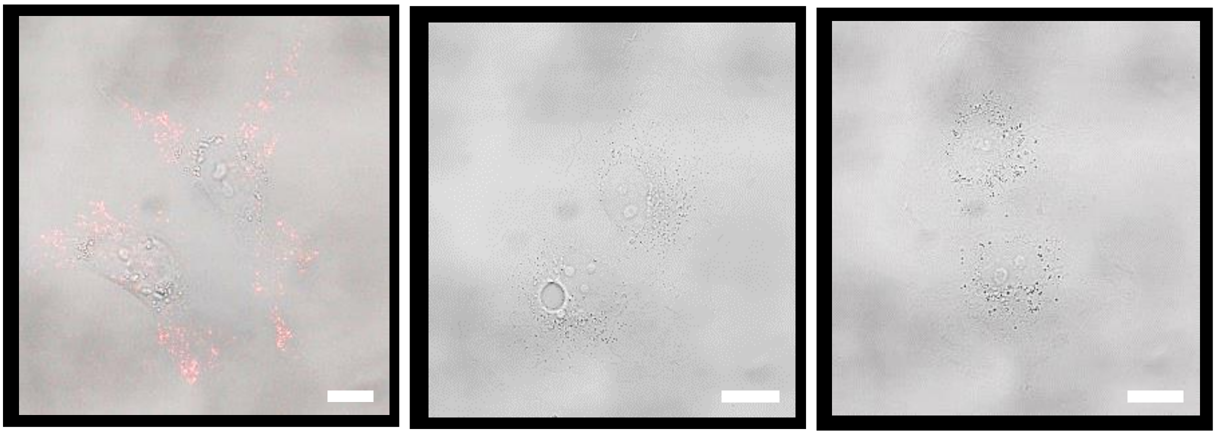

Figure S9. Confocal microscopic images of Langerin+, DC-SIGN+ and wild type COS-7 cells (grey) after the treatment of Biv-Glc2NTs-13-Atto647 (red) for 10 min (scale bar: 20 μm).

[1] K. L. Dueholm, M. Egholm, C. Behrens, L. Christensen, H. F. Hansen, T. Vulpius, K. H. Petersen, R. H. Berg, P. E. Nielsen, O. Buchardt, *The Journal of Organic Chemistry* **1994**, *59*, 5767-5773.

[2] M. Zhou, I. Ghosh, *Organic Letters* **2004**, *6*, 3561-3564.

[3] C. Scheibe, A. Bujotzek, J. Dernedde, M. Weber, O. Seitz, *Chem. Sci.* **2011**, *2*.

[4] E. C. Wamhoff, J. Schulze, L. Bellmann, M. Rentzsch, G. Bachem, F. F. Fuchsberger, J. Rademacher, M. Hermann, B. Del Frari, R. van Dalen, D. Hartmann, N. M. van Sorge, O. Seitz, P. Stoitzner, C. Rademacher, *ACS Cent. Sci.* **2019**, *5*, 808-820.

[5] M. Martinez-Bailen, E. Jimenez-Ortega, A. T. Carmona, I. Robina, J. Sanz-Aparicio, D. Talens-Perales, J. Polaina, C. Matassini, F. Cardona, A. J. Moreno-Vargas, *Bioorg. Chem.* **2019**, *89*, 103026.

[6] aOriginLab*, Vol. 9.1; 2015*, **9.1; 2015**; bMestrelab, **11.0.2; 2016**.

[7] F. Micheel, E. Michaelis, *Chemische Berichte* **1958**, *91*, 188-194.

[8] U. Ellervik, G. Magnusson, *Carbohydr. Res.* **1996**, *280*, 251-260.

[9] J. Aretz, E. C. Wamhoff, J. Hanske, D. Heymann, C. Rademacher, *Front Immunol* **2014**, *5*, 323.

[10] E. C. Wamhoff, J. Hanske, L. Schnirch, J. Aretz, M. Grube, D. Varon Silva, C. Rademacher, *ACS Chem. Biol.* **2016**, *11*, 2407-2413.
